# Robustness of meta-analyses in finding gene × environment interactions

**DOI:** 10.1371/journal.pone.0171446

**Published:** 2017-03-31

**Authors:** Gang Shi, Arye Nehorai

**Affiliations:** 1 State Key Laboratory of Integrated Services Networks, Xidian University, Xi’an, Shaanxi, China; 2 The Preston M. Green Department of Electrical and Systems Engineering, Washington University in St. Louis, St. Louis, Missouri, United States of America; University of Texas School of Public Health, UNITED STATES

## Abstract

Meta-analyses that synthesize statistical evidence across studies have become important analytical tools for genetic studies. Inspired by the success of genome-wide association studies of the genetic main effect, researchers are searching for gene × environment interactions. Confounders are routinely included in the genome-wide gene × environment interaction analysis as covariates; however, this does not control for any confounding effects on the results if covariate × environment interactions are present. We carried out simulation studies to evaluate the robustness to the covariate × environment confounder for meta-regression and joint meta-analysis, which are two commonly used meta-analysis methods for testing the gene × environment interaction or the genetic main effect and interaction jointly. Here we show that meta-regression is robust to the covariate × environment confounder while joint meta-analysis is subject to the confounding effect with inflated type I error rates. Given vast sample sizes employed in genome-wide gene × environment interaction studies, non-significant covariate × environment interactions at the study level could substantially elevate the type I error rate at the consortium level. When covariate × environment confounders are present, type I errors can be controlled in joint meta-analysis by including the covariate × environment terms in the analysis at the study level. Alternatively, meta-regression can be applied, which is robust to potential covariate × environment confounders.

## Introduction

Genome-wide association studies (GWASs) have achieved considerable success in recent years. Approximately 24,000 associations between single nucleotide polymorphisms (SNPs) and complex diseases or traits have been identified [1]. For blood pressure or hypertension traits, more than 60 genomic loci have been discovered, most of which are novel [2]. These findings provide insight into the pathogenesis of common complex diseases, potential targets for pharmacotherapy, as well as clues for precision medicine [3]. Because of the large number of SNPs under statistical testing and small effect sizes for loci of interest, collaborative consortia have been established by studies to achieve sample sizes necessary for discovery and replication. Genetic main effects have been estimated in contributing studies, the results of which were then combined at the consortium level. Meta-analyses methods for synthesizing statistical evidence across studies have become important analytical tools for genetic studies [4].

Inspired by the success of GWASs of genetic main effect, researchers are looking for evidence of gene × environment (G×E) interactions [5]. The genome-wide gene × environment interaction (GWEI) study turned out to be more challenging [6] and the results are limited. In a GWEI study of genetic interactions with body mass index (BMI) for fasting insulin and glucose, six novel loci were identified with genome-wide significance [7]. The regression coefficients of the SNP main effects and SNP×BMI interactions were estimated in studies, while accounting for their covariance. Joint meta-analysis (JMA) was used to test the SNP main effects and interactions simultaneously [8, 9]. In another JMA of SNP and SNP × smoking interaction for pulmonary function, three novel loci were identified [10]. In a genome-wide assessment of SNP × age interactions for blood pressure traits, two novel loci were discovered [11]. Samples were first stratified into six 10-year age bins in each study. The SNP main effects were estimated in strata, as in GWAS. Meta-regression (MR) was then used at the consortium level to conduct the joint test of SNP main effects and SNP × age interactions [12]. More recently, a genome-wide study of SNP × age, SNP × gender, and SNP × age × gender interactions on body size and shape traits was reported [13]. Study-specific GWASs were conducted by four strata (men < 50 y, men > 50 y, women < 50 y, and women > 50 y) and meta-analyses of SNP main effects were also carried out by strata. Four significant loci were novel in the SNP × age interaction test for BMI when comparing meta-results between age strata, and 17 were novel in the SNP × gender interaction test for the waist-to-hip ratio. Although BMI, age, and gender are not environmental in a strict sense, they represent particular genetic and environmental contexts that modulate genetic effects.

From a methodologic perspective, GWEI studies are subject to different types of confounding factors [6, 14–16]. In particular, covariate × environment (C×E) interactions are routinely ignored in current GWEI analyses, which might confound the results [15]. In this work, we carried out simulation studies to determine robustness of the MR and JMA, two commonly used meta-analysis methods for testing interactions or genetic main effects and interactions jointly, to the confounding effects due to the lack of awareness the of C×E interactions.

## Methods

### Meta-regression

MR is a robust, general, and powerful meta-analysis method for conducting GWEI analysis at the consortium level [12] for the following reasons: 1) MR can be used in cases with dichotomous or continuous environmental exposures; 2) MR can be used for testing interactions only, joint testing of genetic main effects and interactions, or testing of marginal genetic main effects; 3) MR can be used to investigate interactions in linear or non-linear forms without requiring additional analyses at the study level; and 4) MR is simple for implementation, so that any study with a conventional GWAS analysis pipeline can contribute to the GWEI analysis.

For continuous environmental exposure, samples are first stratified into groups according to the environmental measurements at each study. SNP main effects for quantitative traits can be estimated at the stratum level using the following linear regression,

$$Y=\beta_{0}+\beta_{\text{G}}G+\beta_{\text{E}}E+\beta_{\text{C}}C+\varepsilon ,$$ (1)

where *Y* is the trait of interest, *G* is the code of the SNP (e.g., the number of reference allele when testing additive genetic effects), *E* is the environmental measurement, and *C* is the covariate. Additional covariates may be included in the model if necessary.

Suppose that ${\hat{\beta }}_{\text{G}i}$ and ${\hat{e}}_{i}$ are the estimated genetic main effect and standard error, respectively, and ${\bar{E}}_{i}$ is the average environmental measurement of the *i*-th stratum, where *i* = 1,2,⋯,*N*, and *N* is the number of strata contributing to the meta-analysis. Linear MR formulates the environment-dependent genetic effect *β*_G_ as follows:

$$\left[\begin{array}{c} {\hat{\beta }}_{\text{G}1}\\ \vdots \\ {\hat{\beta }}_{\text{G}N} \end{array}\right]=\gamma_{0}\left[\begin{array}{c} 1\\ \vdots \\ 1 \end{array}\right]+\gamma_{1}\left[\begin{array}{c} {\bar{E}}_{1}\\ \vdots \\ {\bar{E}}_{N} \end{array}\right]+\left[\begin{array}{c} \epsilon_{1}\\ \vdots \\ \epsilon_{N} \end{array}\right],$$ (2)

where *ϵ*_*i*_ follows a normal distribution with a zero mean and variance ${\hat{e}}_{i}^{2}$ for *i* = 1,2,⋯,*N*. The MR coefficients **γ** = [*γ*_0_,*γ*_1_]^T^ and its covariance can be estimated as $\hat{\gamma }={\left(A^{\text{T}}\Psi^{-1}A\right)}^{-1}A^{\text{T}}\Psi^{-1}\beta$ and Ω = (*A*^T^Ψ^−1^*A*)^−1^, where $\beta ={\left[{\hat{\beta }}_{\text{G}1},\;\cdots ,{\hat{\beta }}_{\text{G}N}\right]}^{\text{T}}$ and

$$\begin{array}{cc} A=\left[\begin{array}{cc} 1 & {\bar{E}}_{1}\\ \vdots & \vdots \\ 1 & {\bar{E}}_{N} \end{array}\right], & \Psi =\left[\begin{array}{ccc} {\hat{e}}_{1}^{2} & \cdots & 0\\ \vdots & \ddots & \vdots \\ 0 & \cdots & {\hat{e}}_{N}^{2} \end{array}\right] \end{array}.$$

The one degree of freedom (1 df) test of interaction is based on the test of slope *γ*_1_. Under the null hypothesis H_0_: *γ*_1_ = 0, test statistics ${\hat{\gamma }}_{1}/\Omega_{2,2}$ follows a Z distribution. The two degrees of freedom (2 df) joint test of genetic main effect and interaction is based on the joint test of intercept *γ*_0_ and slope *γ*_1_. Under the null hypothesis H_0_: *γ*_0_ = *γ*_1_ = 0, test statistics ${\hat{\gamma }}^{\text{T}}\Omega^{-1}\hat{\gamma }$ follows a 2 df chi-square distribution.

The marginal genetic main effect can be tested as follows:

$$\left[\begin{array}{c} {\hat{\beta }}_{\text{G}1}\\ \vdots \\ {\hat{\beta }}_{\text{G}N} \end{array}\right]=\gamma_{0}\left[\begin{array}{c} 1\\ \vdots \\ 1 \end{array}\right]+\left[\begin{array}{c} \epsilon_{1}\\ \vdots \\ \epsilon_{N} \end{array}\right].$$ (3)

In this case, the solution is equivalent to that of the inverse variance meta-analysis method and the test is based on the test of *γ*_0_.

### Joint meta-analysis

For JMA, GWEI analyses are carried out at the study level using linear regression [8, 9], which estimates genetic main effects and interaction jointly, as follows:

$$Y=\beta_{0}+\beta_{\text{G}}G+\beta_{\text{I}}G\times E+\beta_{\text{E}}E+\beta_{\text{C}}C+\varepsilon .$$ (4)

Each study reports the estimated genetic effect ${\hat{\beta }}_{\text{G}i}$, interaction effect ${\hat{\beta }}_{\text{I}i}$ and the covariance matrix ${\hat{\Sigma }}_{i}$, where *i* = 1,2,⋯,*M*, and *M* is the number of studies. At the consortium level, JMA combines statistical evidence by solving the following multivariate generalized least squares equation:

$$\left[\begin{array}{c} {\hat{\beta }}_{\text{G}1}\\ \begin{array}{c} {\hat{\beta }}_{\text{I}1}\\ \vdots \\ {\hat{\beta }}_{\text{G}M} \end{array}\\ {\hat{\beta }}_{\text{I}M} \end{array}\right]=\left[\begin{array}{cc} \begin{array}{c} 1\\ 0 \end{array} & \begin{array}{c} 0\\ 1 \end{array}\\ \vdots & \vdots \\ \begin{array}{c} 1\\ 0 \end{array} & \begin{array}{c} 0\\ 1 \end{array} \end{array}\right]\left[\begin{array}{c} \beta_{\text{G}}\\ \beta_{\text{I}} \end{array}\right]+\left[\begin{array}{c} \epsilon_{1}\\ \vdots \\ \epsilon_{M} \end{array}\right],$$ (5)

where ***ϵ***_*i*_ follows a bivariate normal distribution with a zero mean and covariance matrix ${\hat{\Sigma }}_{i}$, *i* = 1,2,⋯,*M*. The estimate of **β** = [β_G_, β_I_]^T^ and its covariance are $\hat{\beta }={\left(W^{\text{T}}\Sigma^{-1}W\right)}^{-1}W^{\text{T}}\Sigma^{-1}b$ and Φ = (*W*^T^Σ^−1^*W*)^−1^, where $b={\left[{\hat{\beta }}_{\text{G}1},\;{\hat{\beta }}_{\text{I}1},\cdots ,{\hat{\beta }}_{\text{G}M},{\hat{\beta }}_{\text{I}M}\right]}^{\text{T}}$ and

$$\begin{array}{ccc} W=\left[\begin{array}{cc} \begin{array}{c} 1\\ 0 \end{array} & \begin{array}{c} 0\\ 1 \end{array}\\ \vdots & \vdots \\ \begin{array}{c} 1\\ 0 \end{array} & \begin{array}{c} 0\\ 1 \end{array} \end{array}\right] & \text{and} & \Sigma =\left[\begin{array}{ccc} {\hat{\Sigma }}_{1} & \cdots & 0\\ \vdots & \ddots & \vdots \\ 0 & \cdots & {\hat{\Sigma }}_{M} \end{array}\right] \end{array}.$$

The interaction test is based on the test statistics ${\hat{\beta }}_{\text{I}}/\Phi_{2,2}$ which follows a Z distribution. The joint test of the genetic main effect and interaction is based on the 2 df chi-square statistics ${\hat{\beta }}^{\text{T}}\Phi^{-1}\hat{\beta }$.

### Covariate × environment confounder

SNPs that are associated with the covariates, but not the trait of interest, may be significant in genetic association studies simply due to the correlations between the trait and covariates. This type of confounding effects can be effectively controlled by including confounders in the regression model when studying genetic main effects. Confounders, such as age, gender, and principal components [17], are routinely included in the GWAS analysis as covariates. However, covariates may confound the test of the G×E effect through interactions between covariates and the environmental variable [15], which are largely ignored in GWEI studies.

Suppose that a quantitative trait *Y* depends on a genetic effect *G*, an environmental variable *E*, a covariate *C*, a G×E interaction, and a C×E interaction, as follows:

$$Y=\beta_{0}+\beta_{\text{G}}G+\beta_{\text{I}}G\times E+\beta_{\text{E}}E+\beta_{\text{C}}C+\beta_{\text{C}\times \text{E}}C\times E+\varepsilon ,$$ (6)

Assume that only the main effect of the covariate is controlled in the G×E interaction analysis, thus association analysis is carried out by solving the following model without accounting for the C×E interaction:

$$Y=\beta_{0}^{*}+\beta_{\text{G}}^{*}G+\beta_{\text{I}}^{*}G\times E+\beta_{\text{E}}^{*}E+\beta_{\text{C}}^{*}C+\varepsilon .$$

In this case, the estimated interaction effect would be biased by the C×E interaction, as follows [15]:

$$\beta_{\text{I}}^{*}=\beta_{\text{I}}+\beta_{\text{C}\times \text{E}}\frac{\sigma_{\text{CG}}}{\sigma_{\text{G}}^{2}}.$$

Here, *σ*_CG_ is the covariance between *C* and *G*, and $\sigma_{\text{G}}^{2}$ is the variance of *G*. Statistical evidence of the interaction effect comes from two sources: the genuine interaction effect, if it exists; and the C×E interaction. Depending on whether signs of *β*_I_ and *β*_C×E_σ_CG_ are the same or opposite, the statistical power of detecting the G×E interaction could be higher or lower. Even though the C×E confounder may enhance the detection under some scenarios, it should be controlled for because the increased statistical evidence comes from the artifact of model miss-specification.

If a null SNP is tested with the miss-specified model, the G×E interaction could be significant if the SNP is confounded with the covariate, therefore causes a false-positive. For example, there might be population stratification in the study samples, and sub-populations might respond differently to the environmental variable, i.e., a population × environment interaction might exist. Including environmental variable and principle components in the analysis will control the environmental and population effects, but not their interaction. For those SNPs showing different allele frequencies among sub-populations, they would demonstrate G×E effects simply due to the uncontrolled population × environment interaction. In the following section, we examined the robustness of MR and JMA to such C×E confounder.

## Results

We conducted simulations to study type I errors and the statistical power of MR and JMA in the presence of a C×E confounding effect. Evaluations of MR and JMA for the case without C×E confounders have been described elsewhere [9, 12]. In brief, type I errors of the two methods are well controlled when the C×E confounder does not exist [9, 12]. The statistical power of MR is slightly less than that of JMA because of the stratification of the environmental variable [12]. For the purpose of comparison, we also included the results from a mega-analysis, which is based on the pooled individual-level data from all contributing studies. Although the mega-analysis is hardly implemented in GWEI studies due to various heterogeneities or consent restrictions of studies, it provides a benchmark to examine the efficiency of the meta-analysis methods [18].

We simulated 50 studies each with 1000 unrelated individuals. The simulation included a continuous environmental variable *E* and a covariate *C*, both of which follow a standard normal distribution, and a quantitative trait *Y*. The trait *Y* relates to the environmental variable *E* and covariate *C* in the following way:

$$Y=\beta_{\text{E}}E+\beta_{\text{C}}C+\beta_{\text{C}\times \text{E}}C\times E+\varepsilon .$$

Environmental term *β*_E_*E* and covariate term *β*_C_*C* each explain 10% of the variation in *Y*. The C×E interaction term accounts for 0.1% of variance of *Y*. The random error ε is also normally distributed. *C*, *E* and ε are generated by the rnom function in R [19], and *β*_E_, *β*_C_, and *β*_C×E_ are chosen as square roots of the variances of the corresponding terms. The error variance is chosen such that the trait variance equals 1. A common SNP is simulated to be confounded with the covariate *C*. The SNP has a minor allele frequency (MAF) *f*_1_ for samples with a *C* < 0 and a *f*_2_ for a C ≥ 0, This mimics the situation that there is population stratification in samples, *C* is a principal component derived from genome-wide markers, and the SNP is population-informative. Clearly, the SNP is under the null hypothesis having no genetic main effect or G×E interaction effect for trait *Y*.

### Robustness of meta-regression to the covariate × environment confounder

When conducting MR analyses, we divided samples into five strata for each study according to the environmental measurements, each consisting of 200 individuals. The genetic main effect of the SNP was estimated using regression model (1). With the estimated genetic main effect, standard error, and average environmental measurement from each stratum, we performed MR analyses of the 1 df interaction test and 2 df joint test based on Eq (2).

In JMA, we employed interaction model (4) for the analyses at the study level. The estimated genetic effects, interaction effects, and the covariance matrix were combined using Eq (5). In mega-analyses, we pooled samples of studies under analyses, and carried out the 1 df interaction test and 2 df joint test based on regression model (4).

**Fig 1:**
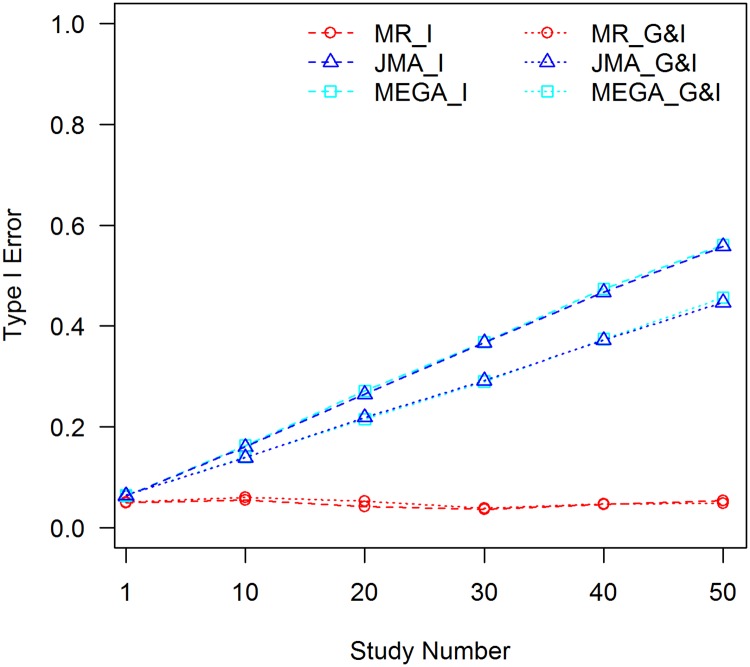
Tests of interaction without controlling for the C×E confounder, *f*_1_ = 0.3, *f*_2_ = 0.1. MR_I: MR test of interaction; MR_G&I: MR joint test of the genetic main effect and interaction; JMA_I: JMA test of interaction; JMA_G&I: JMA joint test of the genetic main effect and interaction; MEGA_I: mega-analysis test of interaction; and MEGA_G&I: mega-analysis joint test of the genetic main effect and interaction.

In this set of analyses, we did not control for the C×E interaction, therefore all analyses are subject to the C×E confounding effect. The analyses were conducted with 1000 replications, empirical type I error rates for the 1 df interaction test and 2 df joint test were evaluated for meta-analyses with 10, 20, 30, 40, and 50 studies. Type I error rates of analyzing a single study were also examined. The results for *f*_1_ = 0.3 and *f*_2_ = 0.1 are shown in Fig 1. Full results for different allele frequencies and different effect sizes of the C×E interaction are presented in the S1–S10 Figs.

**Fig 2:**
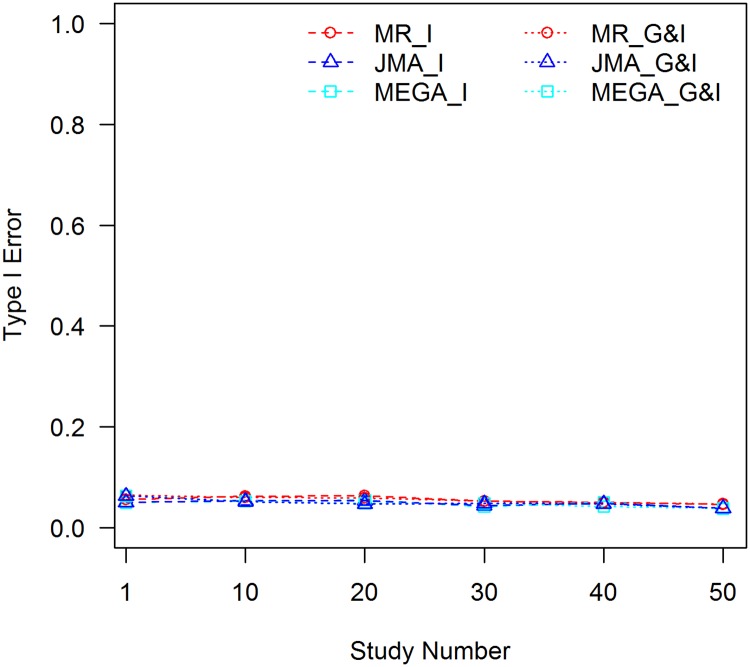
Tests of interaction without controlling for the C×E confounder, *f*_1_ = *f*_2_ = 0.3. MR_I: MR test of interaction; MR_G&I: MR joint test of the genetic main effect and interaction; JMA_I: JMA test of interaction; JMA_G&I: JMA joint test of the genetic main effect and interaction; MEGA_I: mega-analysis test of interaction; and MEGA_G&I: mega-analysis joint test of the genetic main effect and interaction.

It can be seen that MR is robust to the C×E confounder. Empirical type I error rates of the interaction test and joint test behave well for different effect sizes of the C×E interaction, extents of confounding between the covariate and SNP, and sample sizes. JMA and mega-analysis, however, are negatively impacted by the C×E confounding effect with inflated type I error rates when *f*_1_ ≠ *f*_2_. The inflation is larger, when the effect size of the C×E interaction is larger, |*f*_1_ − *f*_2_| is increased, or the sample size becomes larger. The inflation shares the same trend for both the interaction test and the joint test. The latter test is slightly less than the former because the 2 df test has a higher threshold for statistical significance. The type I error rates for JMA and the mega-analysis were close for all scenarios, suggesting that all of the C×E confounding effects are carried over from the study level to the consortium level and included in the meta-statistics. When *f*_1_ equals *f*_2_, type I error rates of the two methods are close to the pre-set nominal value of 0.05 because the SNP is not confounded with the covariate, see Fig 2.

**Fig 3:**
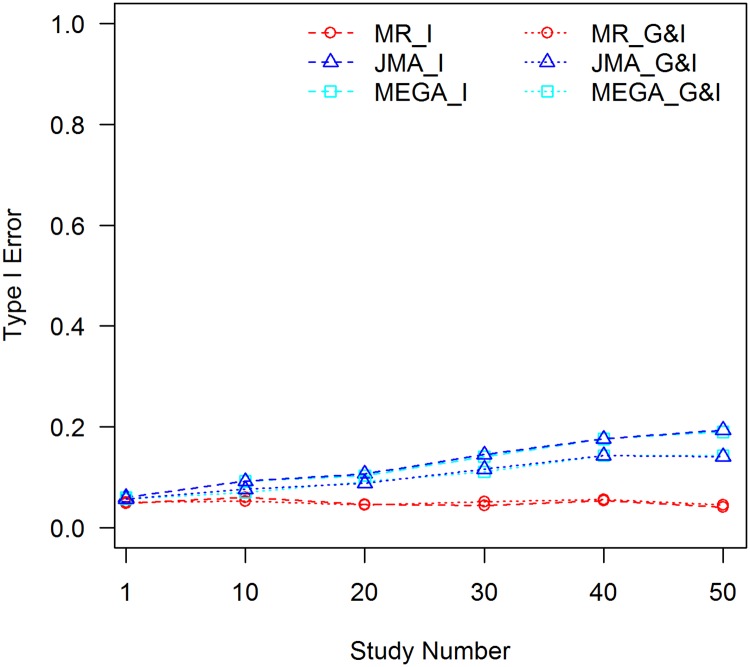
Tests of interaction without controlling for the C×E confounder, *f*_1_ = 0.3, *f*_2_ = 0.1; confounder is present in half of the studies. MR_I: MR test of interaction; MR_G&I: MR joint test of the genetic main effect and interaction; JMA_I: JMA test of interaction; JMA_G&I: JMA joint test of the genetic main effect and interaction; MEGA_I: mega-analysis test of interaction; and MEGA_G&I: mega-analysis joint test of the genetic main effect and interaction.

In GWEI analyses, contributing studies are commonly from different types of designs and with different sampling methods, and not all studies may experience the C×E confounding effect. We carried out another set of simulations, in which half of the studies were assumed to have been subjected to the C×E confounding effect, whereas the others were with *β*_C×E_ set to zero. The results for *f*_1_ = 0.3 and *f*_2_ = 0.1 are shown in Fig 3, and more results are presented in S11–S15 Figs. Obviously, the inflation of type I errors for JMA becomes smaller compared with that in Fig 1. This is because only the studies with C×E effects contributed to the inflation.

### Robustness of analyses of genetic main effects

**Fig 4:**
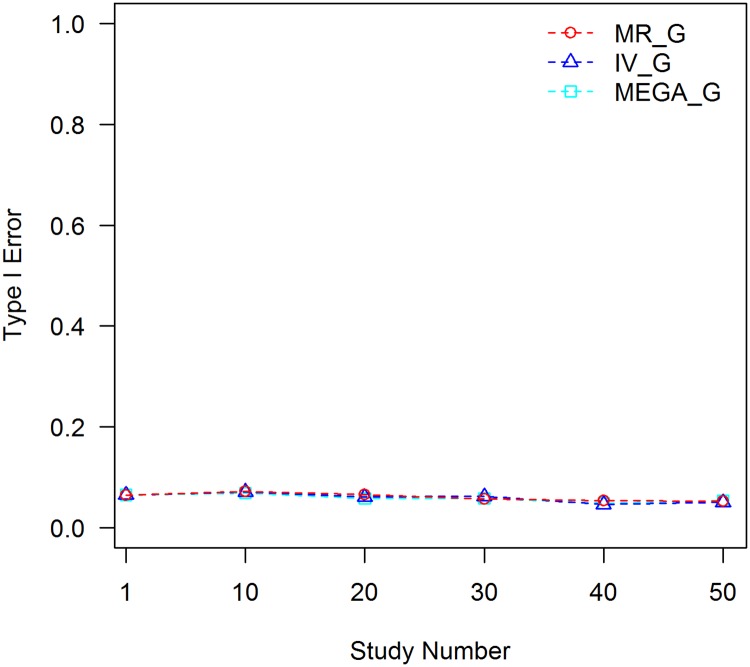
Tests of genetic main effect without controlling for the C×E confounder, *f*_1_ = 0.3, *f*_2_ = 0.1. MR_G: MR test of the genetic main effect; IV_G: inverse variance test of the genetic main effect; and MEGA_G: mega-analysis test of the genetic main effect.

Based on the same simulated data sets, we evaluated the robustness of testing the genetic main effects to the C×E confounder. For MR, the genetic main effect was synthesized using model (3) without additional analyses at the study level. Because JMA does not provide a test of marginal genetic main effects, we compared with the results from an inverse variance meta-analysis method. We estimated genetic main effects of the SNP at each study using model (1). The estimated genetic main effect and standard error were combined with the inverse variance meta-analysis method. Mega-analysis of the genetic main effects was also carried out based on model (1). Empirical type I error rates for testing the SNP main effect are shown in Fig 4.

All of the three analyses were robust to the C×E confounding effect. Although the SNP is confounded with covariate *C* and C×E interaction exists for the simulated trait, it appears that including *C* in the model is sufficient to control for all of the confounding effects. This agrees with the experiences from GWAS, in which all potential interactions are routinely ignored in the analysis of the genetic main effects.

### Accounting for the covariate × environment interaction

**Fig 5:**
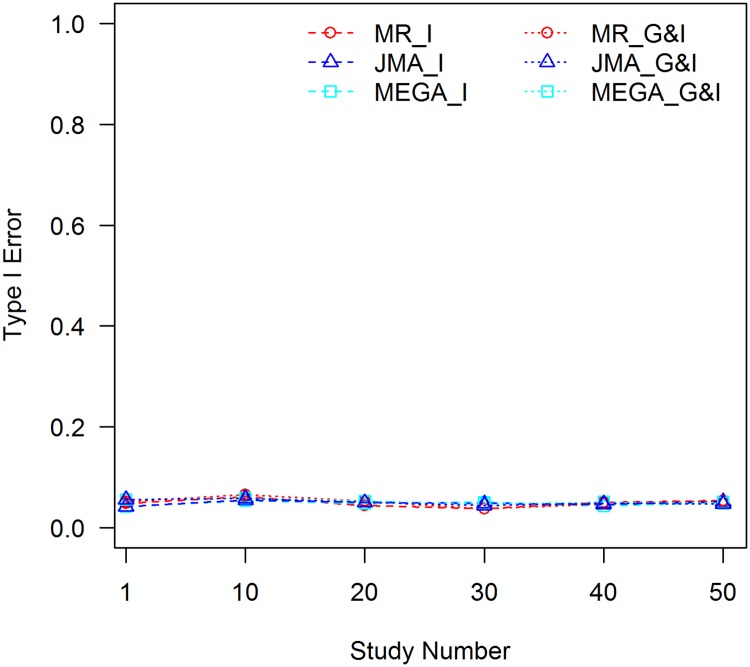
Tests of interaction with controlling for the C×E confounder, *f*_1_ = 0.3, *f*_2_ = 0.1. MR_I: MR test of interaction; MR_G&I: MR joint test of the genetic main effect and interaction; JMA_I: JMA test of interaction; JMA_G&I: JMA joint test of the genetic main effect and interaction; MEGA_I: mega-analysis test of interaction; and MEGA_G&I: mega-analysis joint test of the genetic main effect and interaction.

To prevent the GWEI study from possible C×E confounding effects, a “simple” solution has been suggested [15], that is, including all potential C×E interaction terms in the analysis. Following this recommendation, we re-analyzed the simulated data sets. For JMA, we added the C×E interaction in the analyses at the study level to control for the confounding effect. For MR, we also added the C×E interaction in the analyses, even though unnecessary. The same term was included in the mega-analysis as well. The results are shown in Fig 5. All type I error rates now behave normally, which proves the effectiveness of the solution. For MR, results with and without including the C×E interaction in the model are about the same.

### Statistical power of meta-analysis methods for testing the gene × environment interaction

In MR, samples are stratified into groups according to the environmental measurements. The analysis of genetic main effects by strata essentially overlooks the information on the G×E interaction within the stratum. Statistical evidence of the interaction comes solely from the differences in genetic main effects across strata. The loss of information could be substantial if variations of the environmental variable are large within the strata, thus diminishing the power of detecting the G×E interaction. This loss of information could be alleviated by choosing a finer stratification scheme.

**Fig 6:**
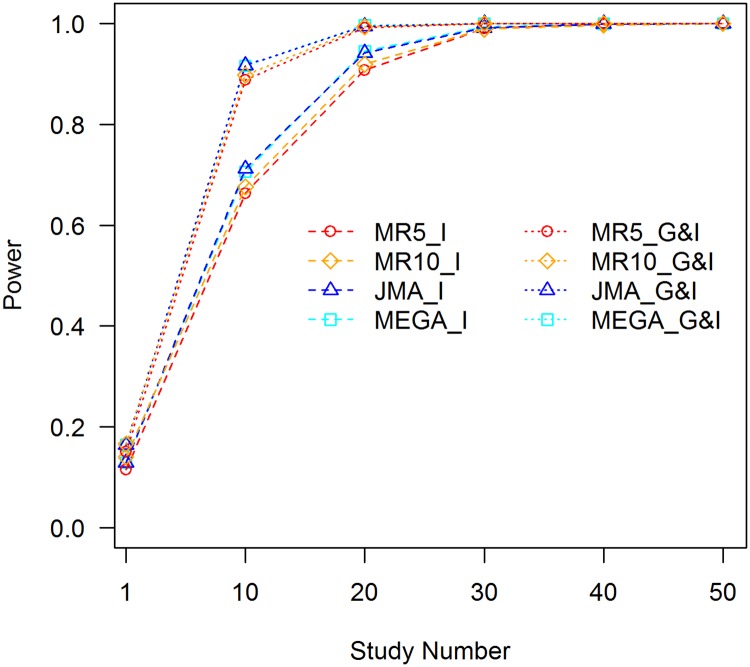
Statistical power of tests of interaction, ${\text{R}}_{G}^{2}=0.05\%,{\text{R}}_{I}^{2}=0.05\%$. MR5_I: MR test of interaction with 5 strata within each study; MR5_G&I: MR joint test of the genetic main effect and interaction with 5 strata within each study; MR10_I: MR test of interaction with 10 strata within each study; MR10_G&I: MR joint test of the genetic main effect and interaction with 10 strata within each study; JMA_I: JMA test of interaction; JMA_G&I: JMA joint test of the genetic main effect and interaction; and MEGA_I: mega-analysis test of interaction; and MEGA_G&I: mega-analysis joint test of the genetic main effect and interaction.

We simulated a causal SNP that is associated with the trait *Y*, as in model (6). The genetic main effect of the SNP, together with the G×E interaction, accounts for 0.1% of trait variance. The environmental effect, covariate effect, and the C×E interaction are the same as in the previous simulation. The SNP has a MAF of 0.3 and the genetic main effect and interaction effect take one-half of the total genetic variance each. MR analyses were conducted in two ways, as follows: dividing the samples of each study into 5 strata; and dividing the samples of each study into 10 strata. The C×E interaction term was included in the JMA and mega-analysis to control for the confounding effect. Fig 6 shows statistical powers of detecting the SNP with the 1 df interaction test and 2 df joint test. More results for different combinations of the genetic main effect and interaction effect are presented in the S26–S30 Figs.

JMA and mega-analysis have essentially the same powers for both the 1 df interaction test and 2 df joint test. Thus, almost all information about the G×E interaction contained in the summary statistics of each study is combined into the meta-statistics efficiently. For the MR of 5 strata at each study, small power losses can be observed for the results with 10 studies. Compared with the mega-analysis, power losses for the 1 df interaction test and 2 df joint test were approximately 6.1% and 3.1%, respectively. When stratifying samples into 10 groups for each study, power losses were reduced to 4.2% and 2.0% for the two tests. The power losses became much smaller when analyzing 20 or more studies.

## Discussion

According to the simulation studies, MR demonstrates robustness to the C×E confounder, while JMA is subject to the confounding effect. There are two major differences between the two approaches. One is that samples were stratified according to the environmental variable and the association analysis is conducted at the stratum level in MR. Because the covariate is adjusted by stratum, most of the C×E variance is eliminated before the meta-analysis. Variation of the environmental exposure within the stratum is small, hence the residual C×E effect is limited. The association analysis of JMA was conducted at the study level with a full range of the environmental variable and was subject to the total effect of the C×E interaction. The other difference is that the genetic main effects were estimated at the stratum level in MR, while both genetic main effect and interaction were estimated in JMA. As shown in Fig 4, estimating marginal genetic main effects is robust to the C×E confounder, as long as the main effect of the covariate is included in the model; however, joint estimation of the genetic main effect and interaction could be confounded by the C×E interaction if it is not accounted for appropriately.

In the current GWEI studies, the genetic main effect and interaction are commonly estimated with covariates included in the model leaving possible C×E interactions uncontrolled. The effect sizes of the C×E interactions may be small and the interactions may not even be significant at the study level; however, given the vast sample sizes of the consortium-type of GWEI studies, small confounding effects could be carried over to the meta-analysis, and final results might suffer from the biases and false positives due to the C×E interactions. In our simulations, with the 0.1% effect size of the C×E interaction and sample size of 1000 for each study, only about 10 out of 50 studies showed statistical significance at the 0.05 level for the C×E interaction effect. The type I error rates increased slightly to 0.06 at the study level for both the interaction test and joint test; however, as shown in Fig 1, if the confounding effect is not properly controlled for, type I error rates increase to 0.56 and 0.45 for the interaction and joint tests, respectively, when results from 50 studies are combined. Therefore, the C×E confounder has to be controlled properly when employing the JMA analysis. Alternatively, a robust method, such as MR, has to be used.

For GWEI studies based on JMA, it is common that multiple covariates are correlated with the trait of interest and multiple C×E interactions may need to be included in the analyses. Otherwise, results would be subject to the C×E confounding effect if one of the C×E confounders is left unaccounted for. In practice, there is usually a lack of information at the consortium level about what C×E interactions should be included in the analysis of which studies due to the heterogeneities of participating studies. In addition, potential C×E interactions may not even be significant in many studies. This results in a difficult decision when preparing the analysis plan. Note that the association analysis of MR is conducted by stratum, and all possible C×E interaction effects are eliminated implicitly at the strata level without requiring such information. It applies well to the case that different studies may need to adjust different sets of C×E interactions.

When evaluating the robustness of meta-analyses to a C×E confounder, we used the covariate that indicates population stratification. We showed the inflation of type I error for population informative SNPs when C×E interaction exists. As demonstrated in many GWAS literature, such population stratification is modest in samples of European ancestry, therefore, the confounding effect of population × environment interaction would be modest as well. For covariates such as age or gender, it is rare to see autosomal SNPs having covariate-dependent allele frequencies. As a result, even if C×E interaction exists, it will not affect the results because SNPs are not confounded with these covariates. This corresponds to the case when *f*_1_ = *f*_2_ as demonstrated in Fig 2.

Robust standard errors are provided in many GWAS software packages, such as ProbABEL [20], in order to avoid the influence of potential heteroscedasticity or outliers. We conducted another set of analyses based on the robust variance and covariance matrix of “HC3” type that is implemented in the sandwich package of R [21]. The results are presented in S31–S35 Figs, which are essentially the same as the results based on the ordinary least squares estimator. Since such robust approach does not address the C×E confounding effect, interaction analyses robust to the heteroscedasticity and outliers without accounting for the C×E interaction are still subject to the inflation of type I error.

Choosing the number of strata for MR is a trade-off among statistical power, analysis complexity and spectrum of allele frequency. Obviously, the number of the analyses at the study level is linearly related to the number of strata. We showed in the Results section that finer stratification scheme provides greater statistical power; however, dividing samples into too many strata will not only increase the analysis burden, but also decrease the sample sizes of the strata. For less-frequent SNPs, the minor allele counts may become so low that stable statistical inference cannot be guaranteed. For example, to ensure minor allele counts to be larger than 20 for SNPs with MAFs larger than 5%, sample sizes of strata should be larger than 200. When applying MR in GWEI studies, a coarse stratification scheme can be chosen for studies with small sample sizes and fine stratification can be used for studies with large sample sizes.

Keller suggested another scenario in which the covariate may confound the interaction analysis [15]. When the covariate is correlated with the environmental variable and the covariate interacts with the SNP, statistical evidence of the G×E interaction could come from the correlation between the covariate and environmental variable. In such cases, the gene × covariate term should be included in the model to produce an unbiased estimate of the G×E effect. Both the MR and JMA are subject to this type of confounding effect; however, any significant SNPs detected because of this kind of confounding effect are under the alternative hypothesis, and are therefore genuine. It is the “interaction with whom” that is under question. Caution has to be taken when interpreting the interactions discovered by usual models without accounting for the gene × covariate (G×C) interaction. *Post hoc* analysis that includes G×C in the model can be carried out for those significant results.

## Supporting information

S1 FigType I error rates of tests of interaction and joint tests of the genetic main effect and interaction without controlling for the C×E confounder, *f*_1_ = 0.3, *f*_2_ = 0.1, and $R_{\text{C}\times \text{E}}^{2}=0.1\%$.(PNG)

S2 FigType I error rates of tests of interaction and joint tests of the genetic main effect and interaction without controlling for the C×E confounder, *f*_1_ = 0.3, *f*_2_ = 0.2, and $R_{\text{C}\times \text{E}}^{2}=0.1\%$.(PNG)

S3 FigType I error rates of tests of interaction and joint tests of the genetic main effect and interaction without controlling for the C×E confounder, *f*_1_ = 0.3, *f*_2_ = 0.3, and $R_{\text{C}\times \text{E}}^{2}=0.1\%$.(PNG)

S4 FigType I error rates of tests of interaction and joint tests of the genetic main effect and interaction without controlling for the C×E confounder, *f*_1_ = 0.3, *f*_2_ = 0.4, and $R_{\text{C}\times \text{E}}^{2}=0.1\%$.(PNG)

S5 FigType I error rates of tests of interaction and joint tests of the genetic main effect and interaction without controlling for the C×E confounder, *f*_1_ = 0.3, *f*_2_ = 0.5, and $R_{\text{C}\times \text{E}}^{2}=0.1\%$.(PNG)

S6 FigType I error rates of tests of interaction and joint tests of the genetic main effect and interaction without controlling for the C×E confounder, *f*_1_ = 0.3, *f*_2_ = 0.1, and $R_{\text{C}\times \text{E}}^{2}=1\%$.(PNG)

S7 FigType I error rates of tests of interaction and joint tests of the genetic main effect and interaction without controlling for the C×E confounder, *f*_1_ = 0.3, *f*_2_ = 0.2, and $R_{\text{C}\times \text{E}}^{2}=1\%$.(PNG)

S8 FigType I error rates of tests of interaction and joint tests of the genetic main effect and interaction without controlling for the C×E confounder, *f*_1_ = 0.3, *f*_2_ = 0.3, and $R_{\text{C}\times \text{E}}^{2}=1\%$.(PNG)

S9 FigType I error rates of tests of interaction and joint tests of the genetic main effect and interaction without controlling for the C×E confounder, *f*_1_ = 0.3, *f*_2_ = 0.4, and $R_{\text{C}\times \text{E}}^{2}=1\%$.(PNG)

S10 FigType I error rates of tests of interaction and joint tests of the genetic main effect and interaction without controlling for the C×E confounder, *f*_1_ = 0.3, *f*_2_ = 0.5, and $R_{\text{C}\times \text{E}}^{2}=1\%$.(PNG)

S11 FigType I error rates of tests of interaction and joint tests of the genetic main effect and interaction without controlling for the C×E confounder, *f*_1_ = 0.3, *f*_2_ = 0.1, and $R_{\text{C}\times \text{E}}^{2}=0.1\%$; confounder is present in half of the studies.(PNG)

S12 FigType I error rates of tests of interaction and joint tests of the genetic main effect and interaction without controlling for the C×E confounder, *f*_1_ = 0.3, *f*_2_ = 0.2, and $R_{\text{C}\times \text{E}}^{2}=0.1\%$; confounder is present in half of the studies.(PNG)

S13 FigType I error rates of tests of interaction and joint tests of the genetic main effect and interaction without controlling for the C×E confounder, *f*_1_ = 0.3, *f*_2_ = 0.3, and $R_{\text{C}\times \text{E}}^{2}=0.1\%$; confounder is present in half of the studies.(PNG)

S14 FigType I error rates of tests of interaction and joint tests of the genetic main effect and interaction without controlling for the C×E confounder, *f*_1_ = 0.3, *f*_2_ = 0.4, and $R_{\text{C}\times \text{E}}^{2}=0.1\%$; confounder is present in half of the studies.(PNG)

S15 FigType I error rates of tests of interaction and joint tests of the genetic main effect and interaction without controlling for the C×E confounder, *f*_1_ = 0.3, *f*_2_ = 0.5, and $R_{\text{C}\times \text{E}}^{2}=0.1\%$; confounder is present in half of the studies.(PNG)

S16 FigType I error rates of tests of the genetic main effect without controlling for the C×E confounder, *f*_1_ = 0.3, *f*_2_ = 0.1, and $R_{\text{C}\times \text{E}}^{2}=0.1\%$.(PNG)

S17 FigType I error rates of tests of the genetic main effect without controlling for the C×E confounder, *f*_1_ = 0.3, *f*_2_ = 0.2, and $R_{\text{C}\times \text{E}}^{2}=0.1\%$.(PNG)

S18 FigType I error rates of tests of the genetic main effect without controlling for the C×E confounder, *f*_1_ = 0.3, *f*_2_ = 0.3, and $R_{\text{C}\times \text{E}}^{2}=0.1\%$.(PNG)

S19 FigType I error rates of tests of the genetic main effect without controlling for the C×E confounder, *f*_1_ = 0.3, *f*_2_ = 0.4, and $R_{\text{C}\times \text{E}}^{2}=0.1\%$.(PNG)

S20 FigType I error rates of tests of the genetic main effect without controlling for the C×E confounder, *f*_1_ = 0.3, *f*_2_ = 0.5, and $R_{\text{C}\times \text{E}}^{2}=0.1\%$.(PNG)

S21 FigType I error rates of the tests of interaction and joint tests of the genetic main effect and interaction with controlling for the C×E confounder, *f*_1_ = 0.3, *f*_2_ = 0.1, and $R_{\text{C}\times \text{E}}^{2}=0.1\%$.(PNG)

S22 FigType I error rates of the tests of interaction and joint tests of the genetic main effect and interaction with controlling for the C×E confounder, *f*_1_ = 0.3, *f*_2_ = 0.2, and $R_{\text{C}\times \text{E}}^{2}=0.1\%$.(PNG)

S23 FigType I error rates of the tests of interaction and joint tests of the genetic main effect and interaction with controlling for the C×E confounder, *f*_1_ = 0.3, *f*_2_ = 0.3, and $R_{\text{C}\times \text{E}}^{2}=0.1\%$.(PNG)

S24 FigType I error rates of the tests of interaction and joint tests of the genetic main effect and interaction with controlling for the C×E confounder, *f*_1_ = 0.3, *f*_2_ = 0.4, and $R_{\text{C}\times \text{E}}^{2}=0.1\%$.(PNG)

S25 FigType I error rates of the tests of interaction and joint tests of the genetic main effect and interaction with controlling for the C×E confounder, *f*_1_ = 0.3, *f*_2_ = 0.5, and $R_{\text{C}\times \text{E}}^{2}=0.1\%$.(PNG)

S26 FigStatistical power of tests of interaction and joint tests of the genetic main effect and interaction, $R_{\text{G}}^{2}=0.1\%,\;R_{\text{I}}^{2}=0\%$, $R_{\text{C}\times \text{E}}^{2}=0.1\%$, and MAF = 0.3.(PNG)

S27 FigStatistical power of tests of interaction and joint tests of the genetic main effect and interaction, $R_{\text{G}}^{2}=0.075\%,\;R_{\text{I}}^{2}=0.025\%$, $R_{\text{C}\times \text{E}}^{2}=0.1\%$, and MAF = 0.3.(PNG)

S28 FigStatistical power of tests of interaction and joint tests of the genetic main effect and interaction, $R_{\text{G}}^{2}=0.05\%,\;R_{\text{I}}^{2}=0.05\%$, $R_{\text{C}\times \text{E}}^{2}=0.1\%$, and MAF = 0.3.(PNG)

S29 FigStatistical power of tests of interaction and joint tests of the genetic main effect and interaction, $R_{\text{G}}^{2}=0.025\%,\;R_{\text{I}}^{2}=0.075\%$, $R_{\text{C}\times \text{E}}^{2}=0.1\%$, and MAF = 0.3.(PNG)

S30 FigStatistical power of tests of interaction and joint tests of the genetic main effect and interaction, $R_{\text{G}}^{2}=0\%,\;R_{\text{I}}^{2}=0.1\%$, $R_{\text{C}\times \text{E}}^{2}=0.1\%$, and MAF = 0.3.(PNG)

S31 FigType I error rates of tests of interaction and joint tests of the genetic main effect and interaction without controlling for the C×E confounder, *f*_1_ = 0.3, *f*_2_ = 0.1, and $R_{\text{C}\times \text{E}}^{2}=0.1\%$; robust variance and covariance are used.(PNG)

S32 FigType I error rates of tests of interaction and joint tests of the genetic main effect and interaction without controlling for the C×E confounder, *f*_1_ = 0.3, *f*_2_ = 0.2, and $R_{\text{C}\times \text{E}}^{2}=0.1\%$; robust variance and covariance are used.(PNG)

S33 FigType I error rates of tests of interaction and joint tests of the genetic main effect and interaction without controlling for the C×E confounder, *f*_1_ = 0.3, *f*_2_ = 0.3, and $R_{\text{C}\times \text{E}}^{2}=0.1\%$; robust variance and covariance are used.(PNG)

S34 FigType I error rates of tests of interaction and joint tests of the genetic main effect and interaction without controlling for the C×E confounder, *f*_1_ = 0.3, *f*_2_ = 0.4, and $R_{\text{C}\times \text{E}}^{2}=0.1\%$; robust variance and covariance are used.(PNG)

S35 FigType I error rates of tests of interaction and joint tests of the genetic main effect and interaction without controlling for the C×E confounder, *f*_1_ = 0.3, *f*_2_ = 0.5, and $R_{\text{C}\times \text{E}}^{2}=0.1\%$; robust variance and covariance are used.(PNG)
