# Supplementary figures and images for: Robustness of meta-analyses in finding gene × environment interactions
Source: PLoS One. 2017 Mar 31;12(3):e0171446. doi: 10.1371/journal.pone.0171446 (PMC5375145; doi:10.1371/journal.pone.0171446)

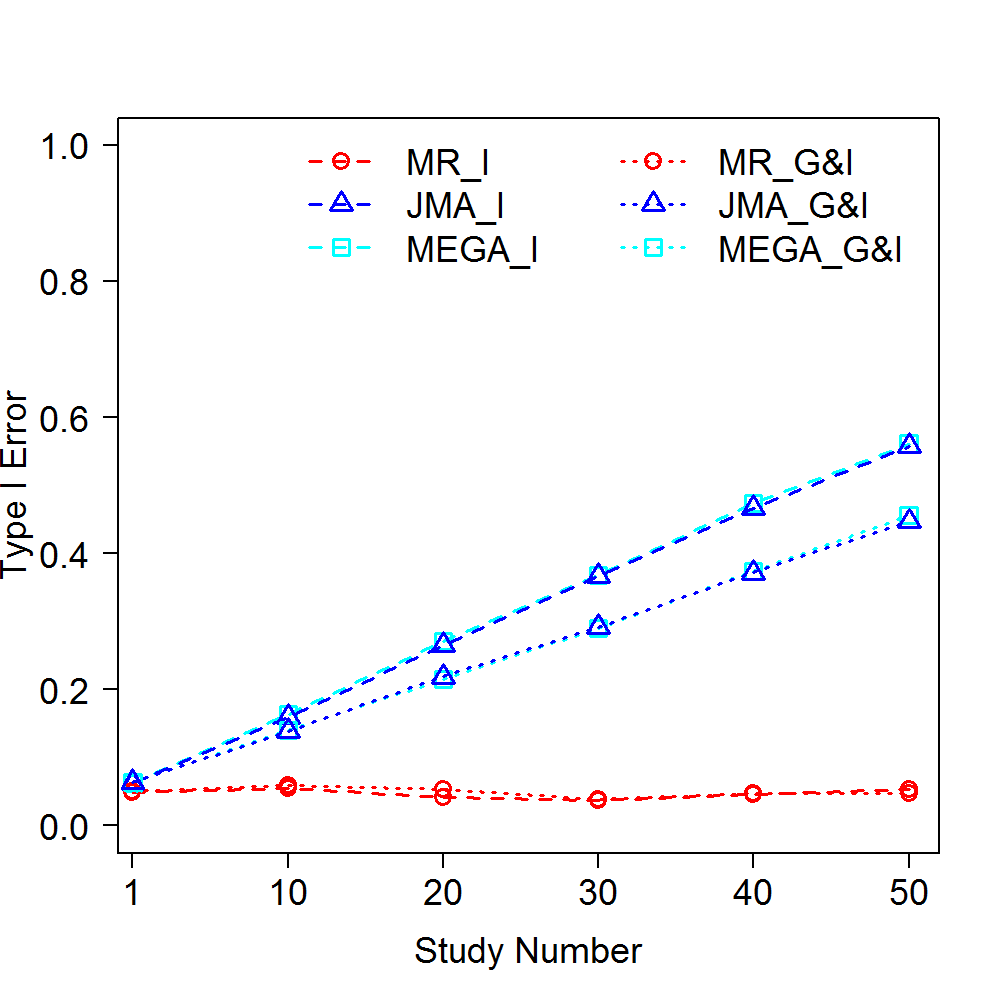

Supplement: S1 Fig — (PNG) [file pone.0171446.s001.png]

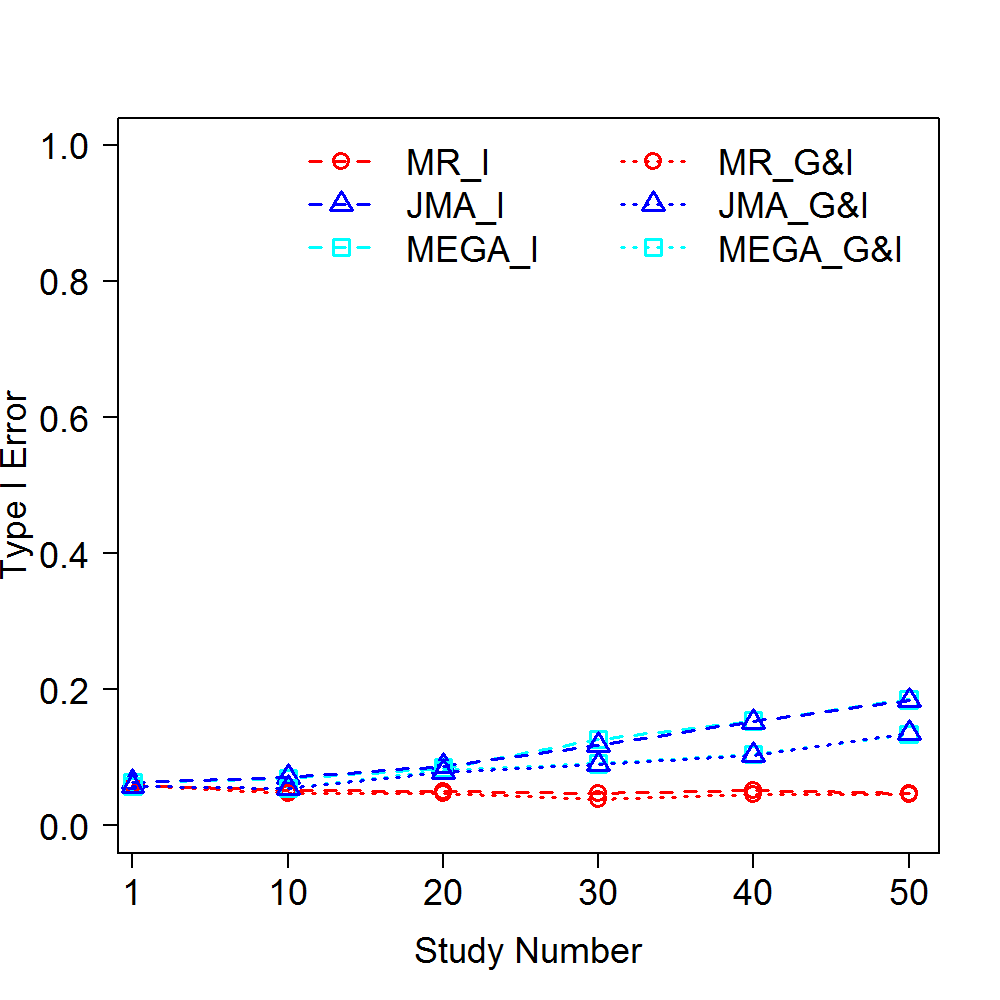

Supplement: S2 Fig — (PNG) [file pone.0171446.s002.png]

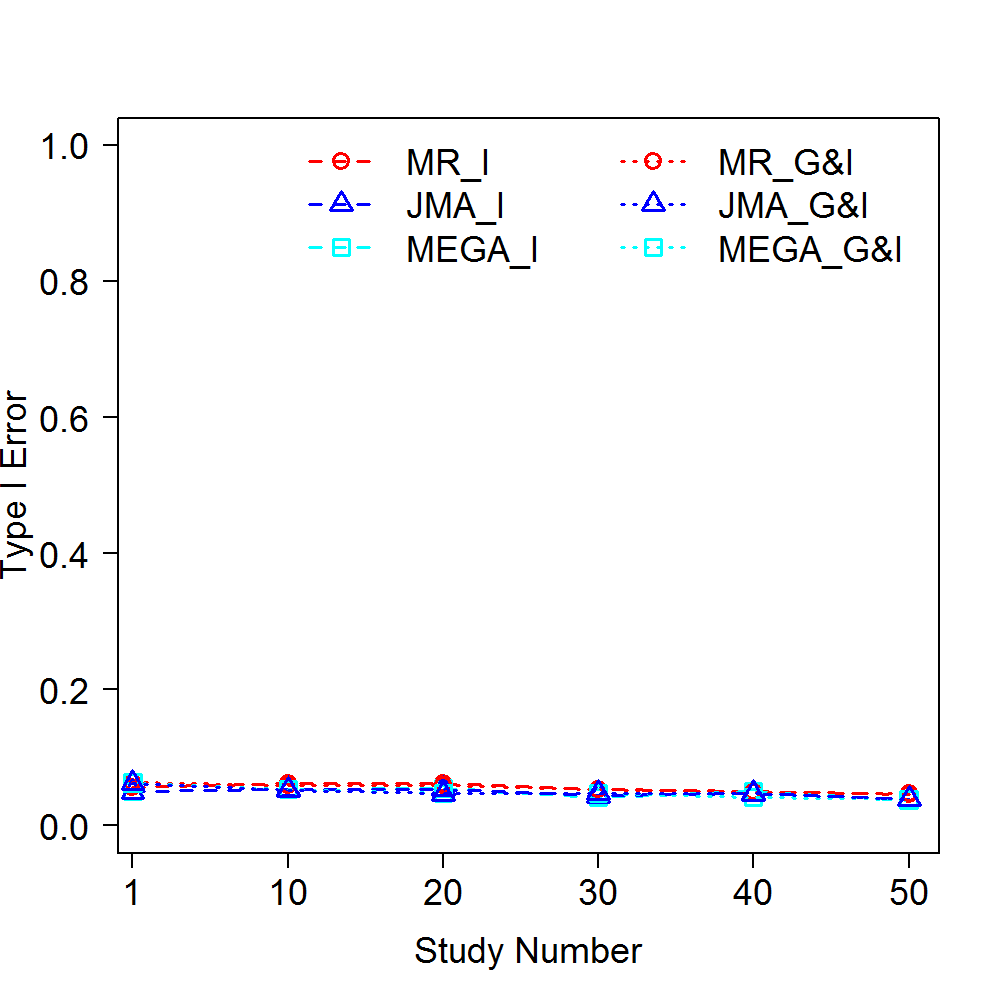

Supplement: S3 Fig — (PNG) [file pone.0171446.s003.png]

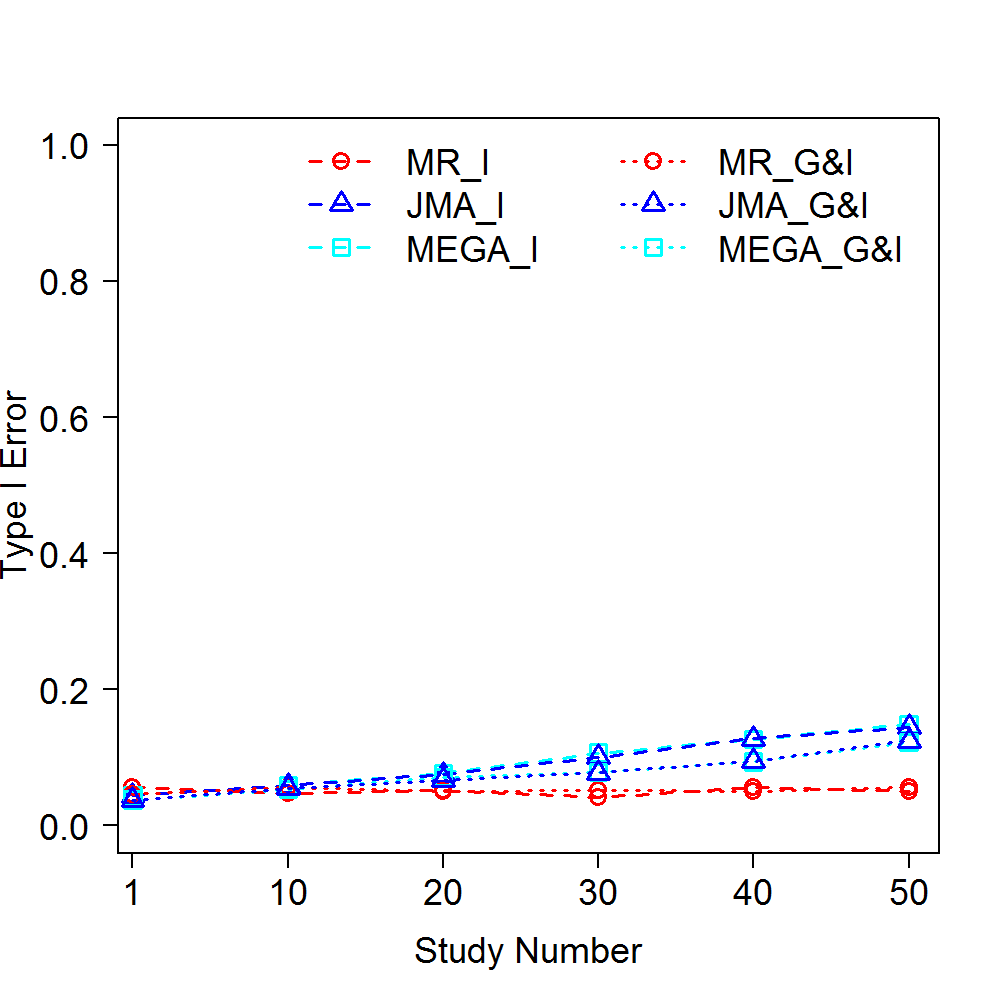

Supplement: S4 Fig — (PNG) [file pone.0171446.s004.png]

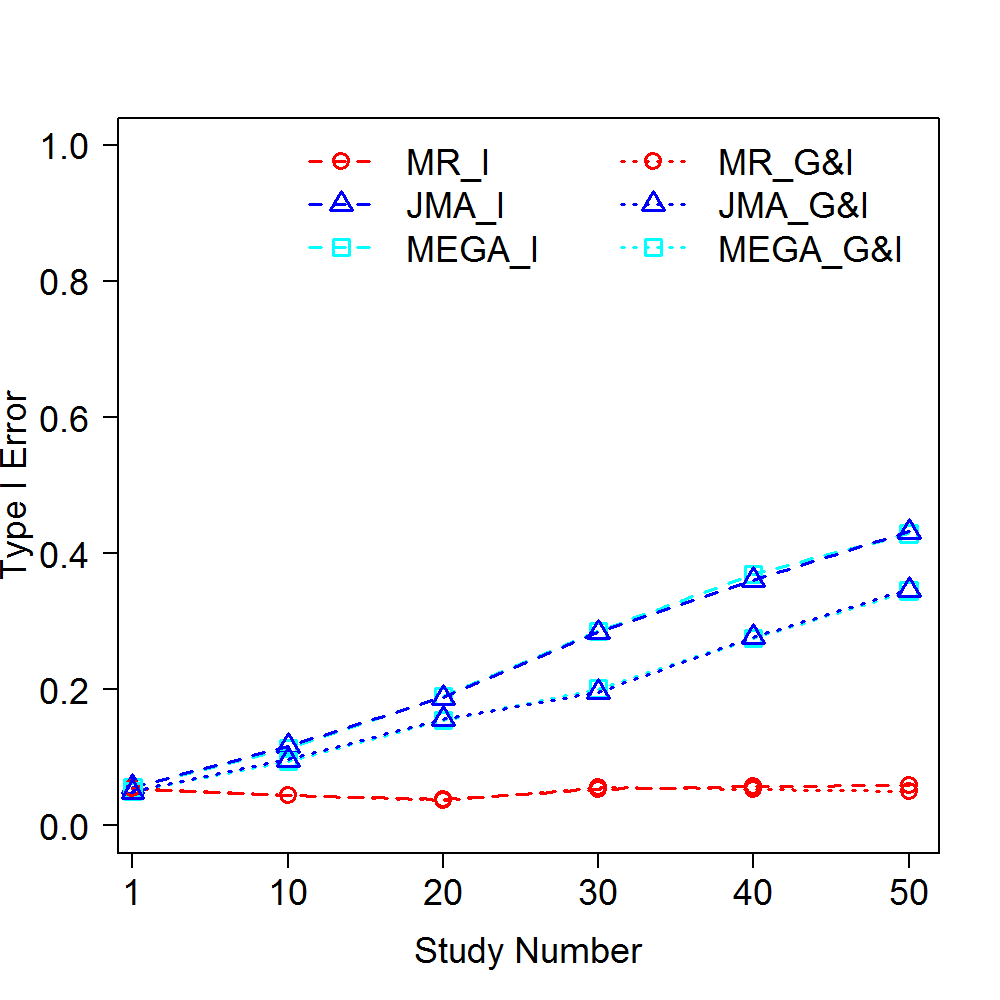

Supplement: S5 Fig — (PNG) [file pone.0171446.s005.png]

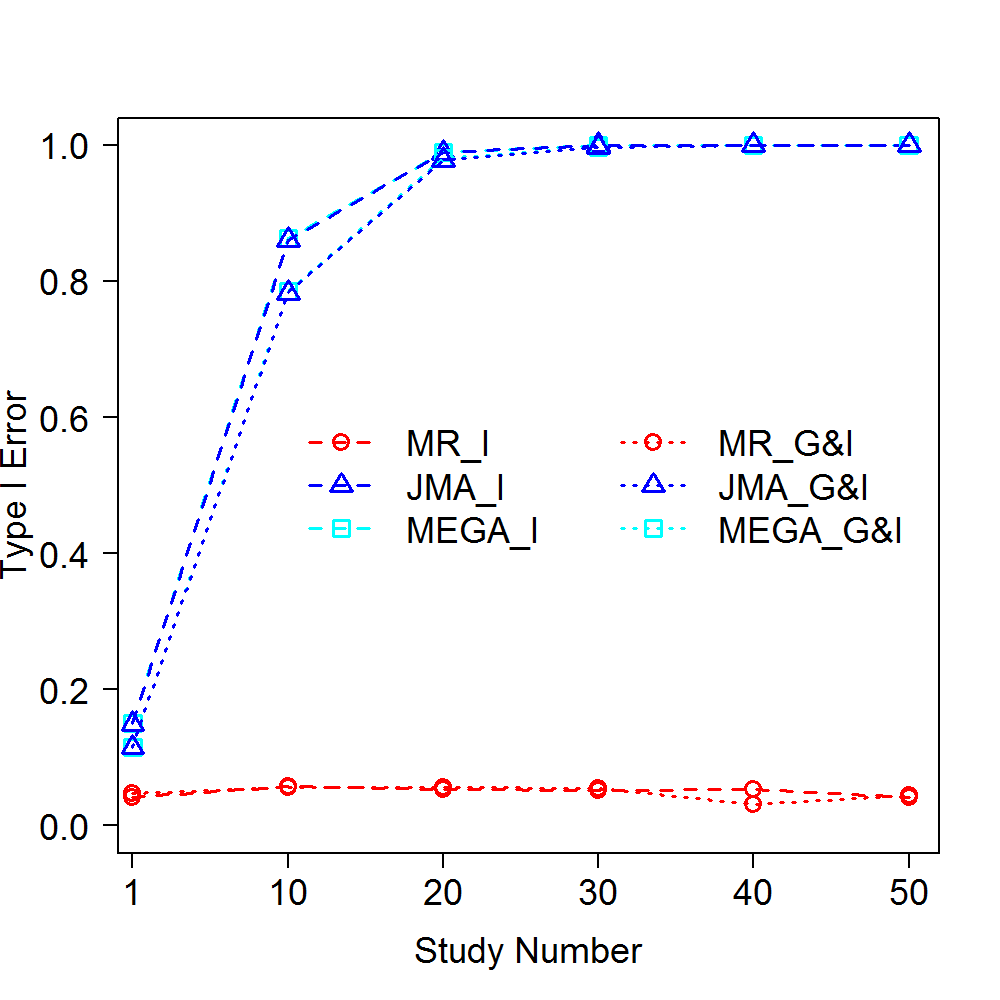

Supplement: S6 Fig — (PNG) [file pone.0171446.s006.png]

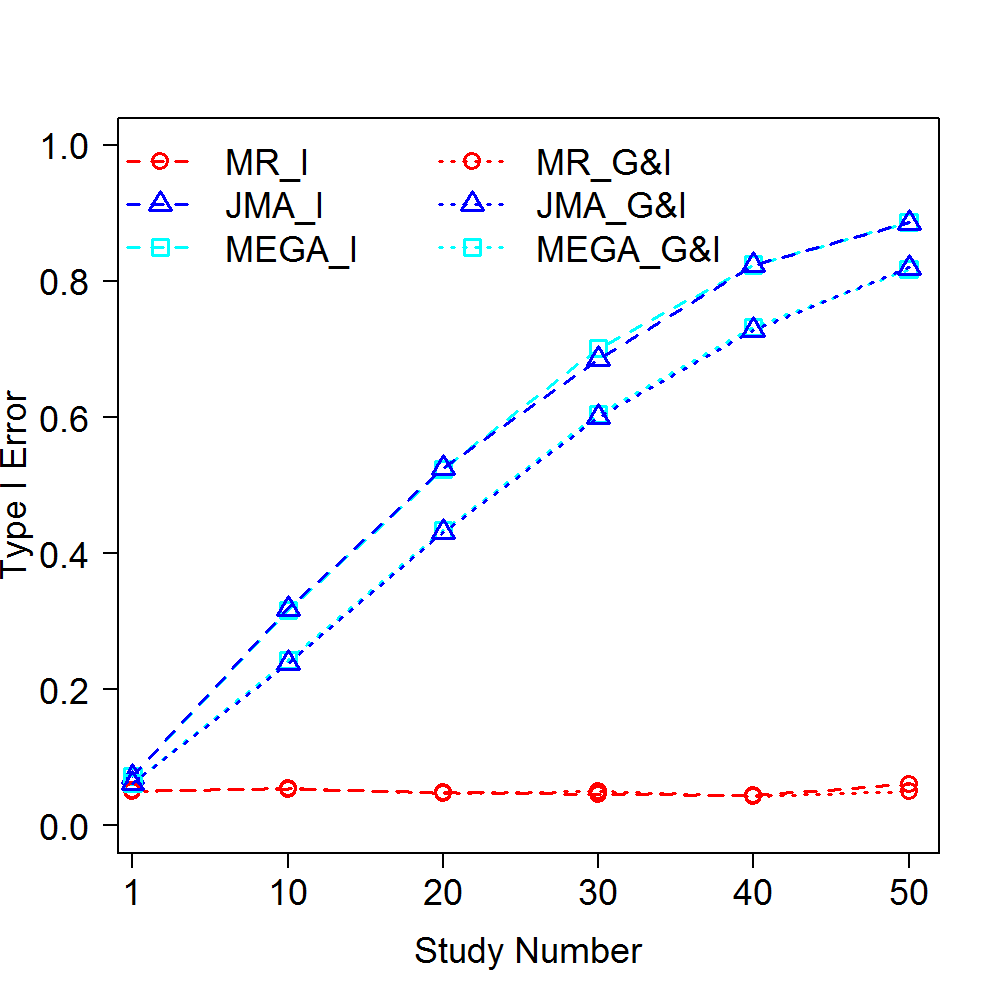

Supplement: S7 Fig — (PNG) [file pone.0171446.s007.png]

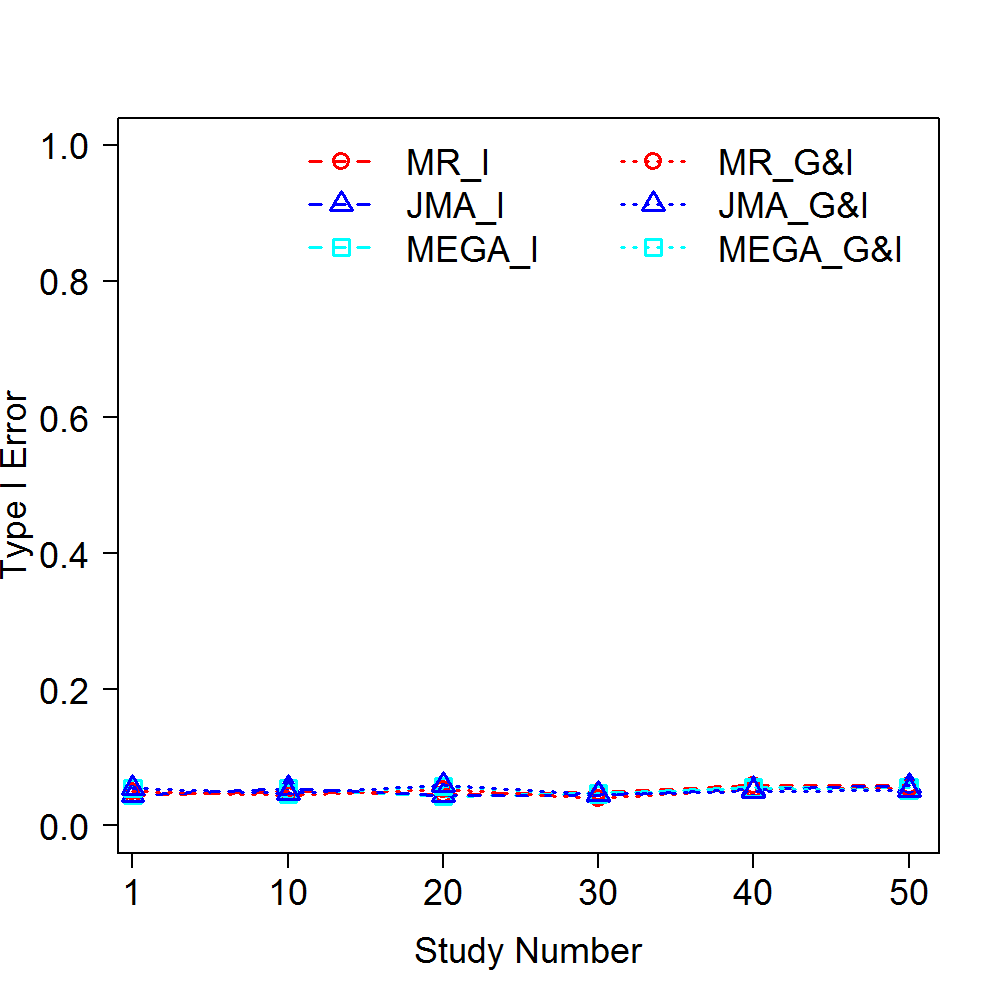

Supplement: S8 Fig — (PNG) [file pone.0171446.s008.png]

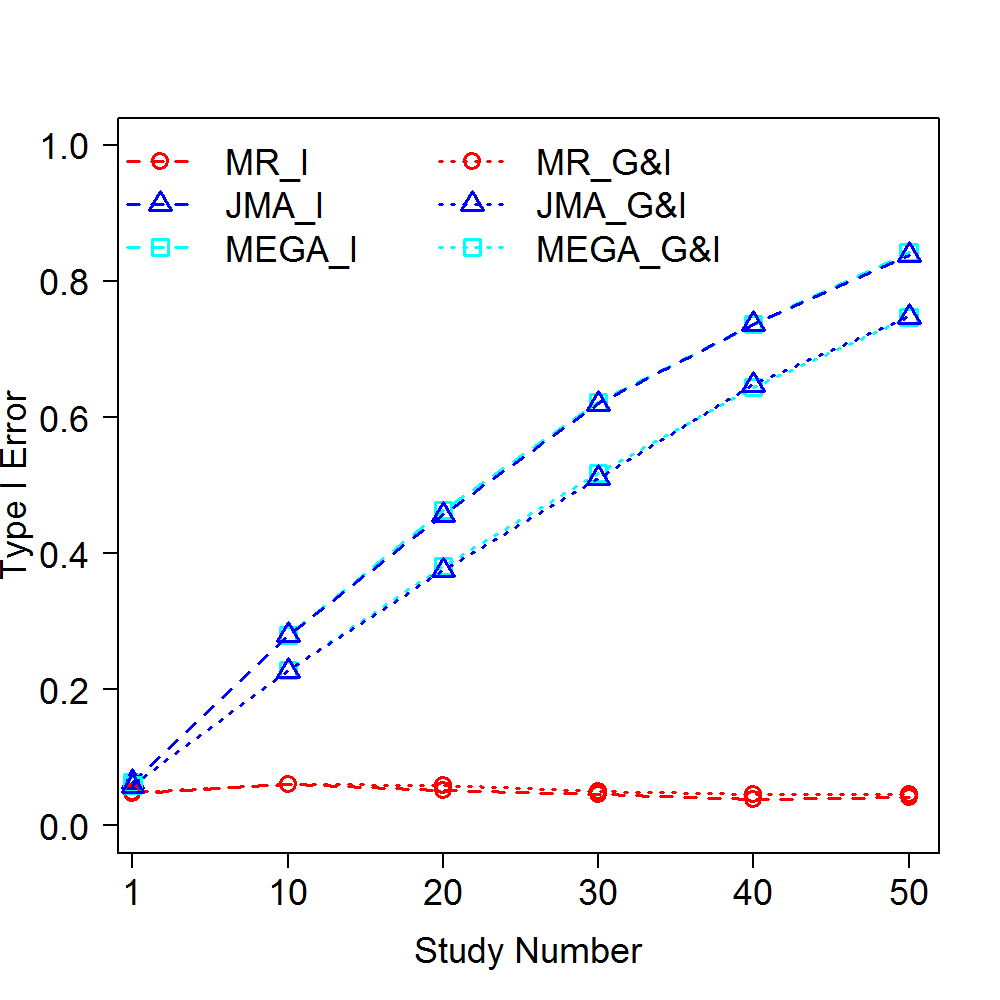

Supplement: S9 Fig — (PNG) [file pone.0171446.s009.png]

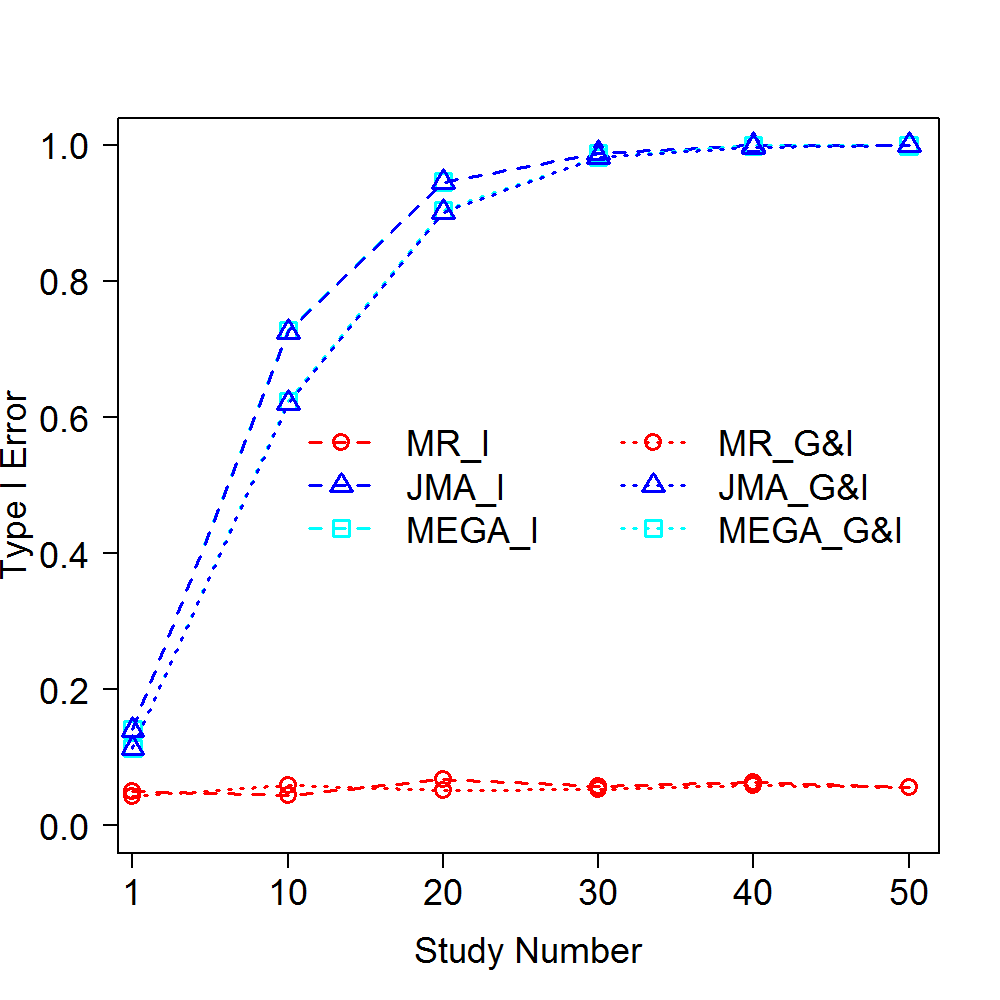

Supplement: S10 Fig — (PNG) [file pone.0171446.s010.png]

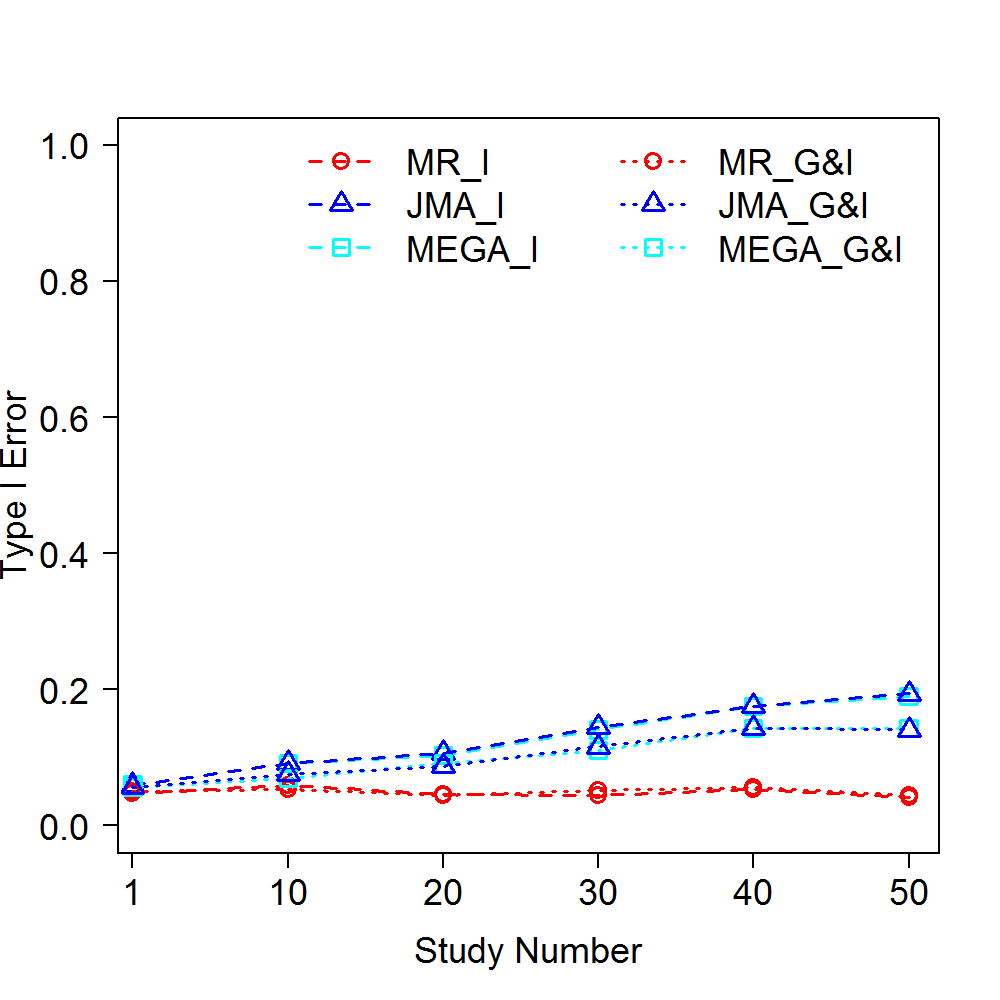

Supplement: S11 Fig — (PNG) [file pone.0171446.s011.png]

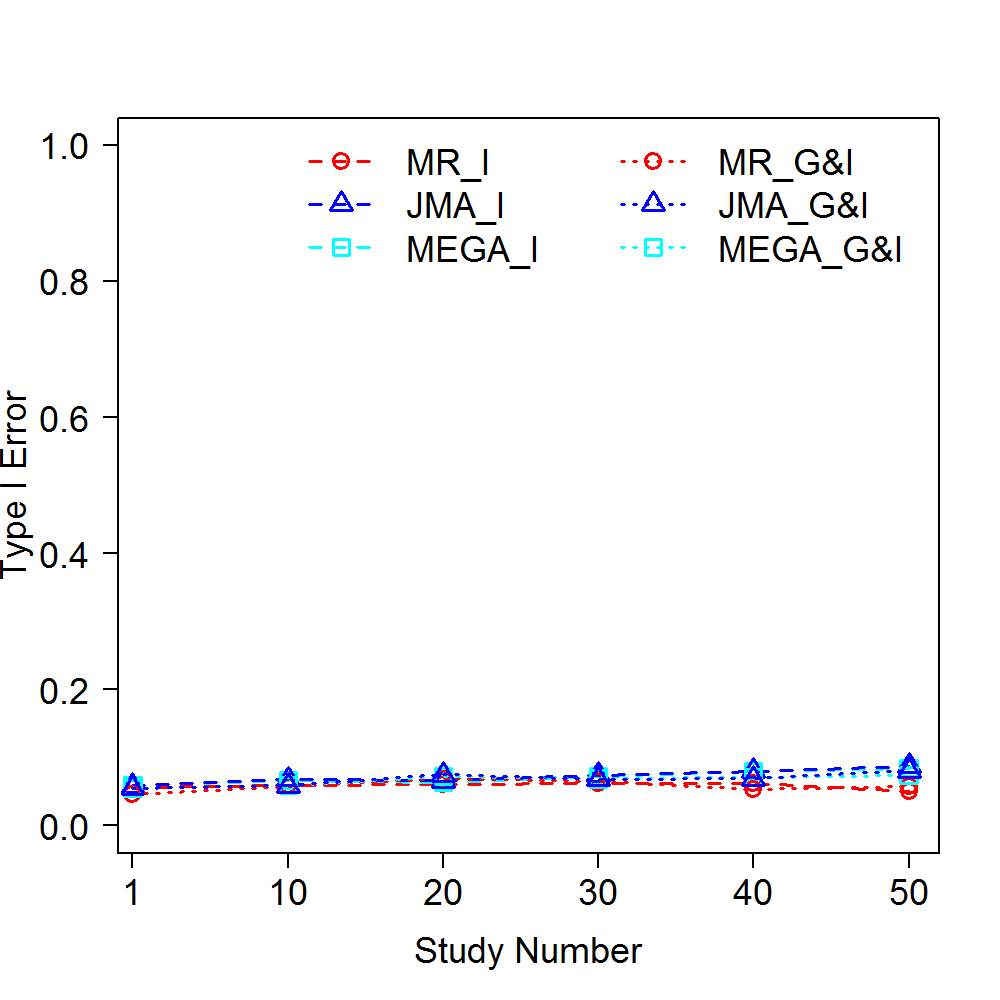

Supplement: S12 Fig — (PNG) [file pone.0171446.s012.png]

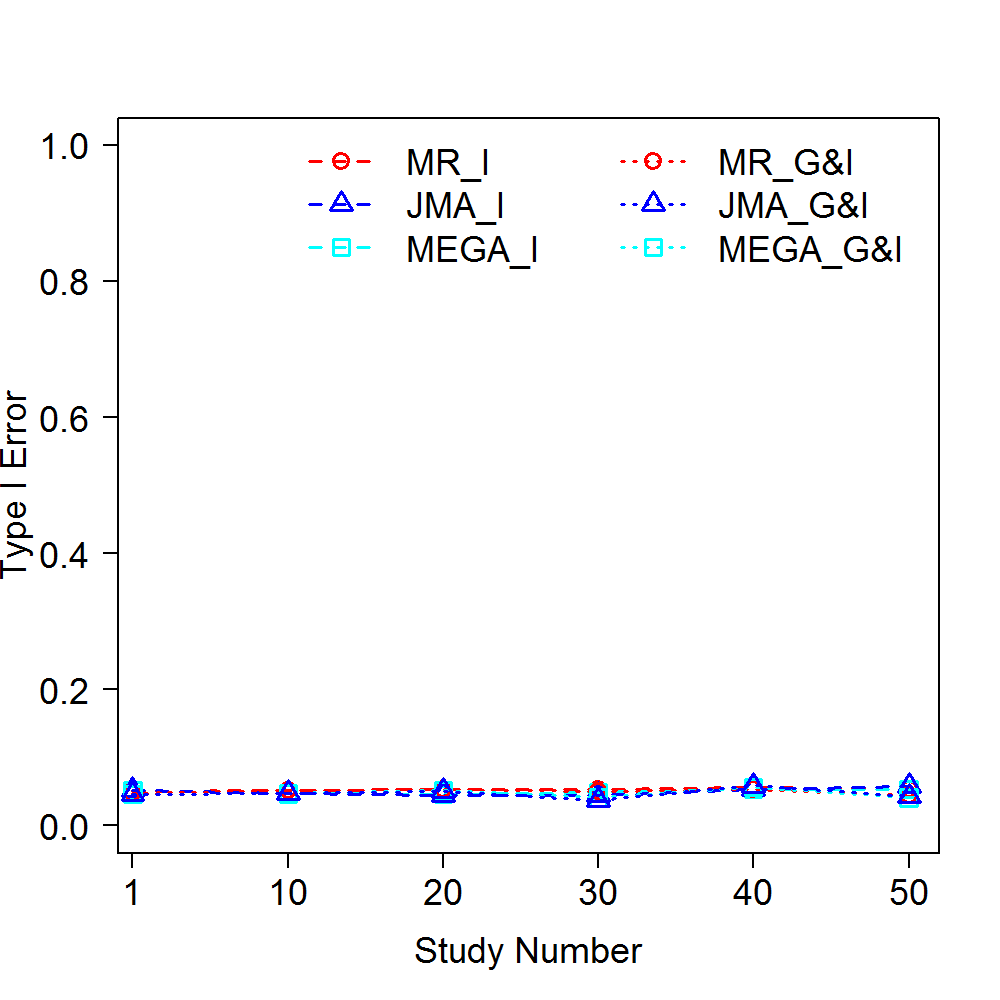

Supplement: S13 Fig — (PNG) [file pone.0171446.s013.png]

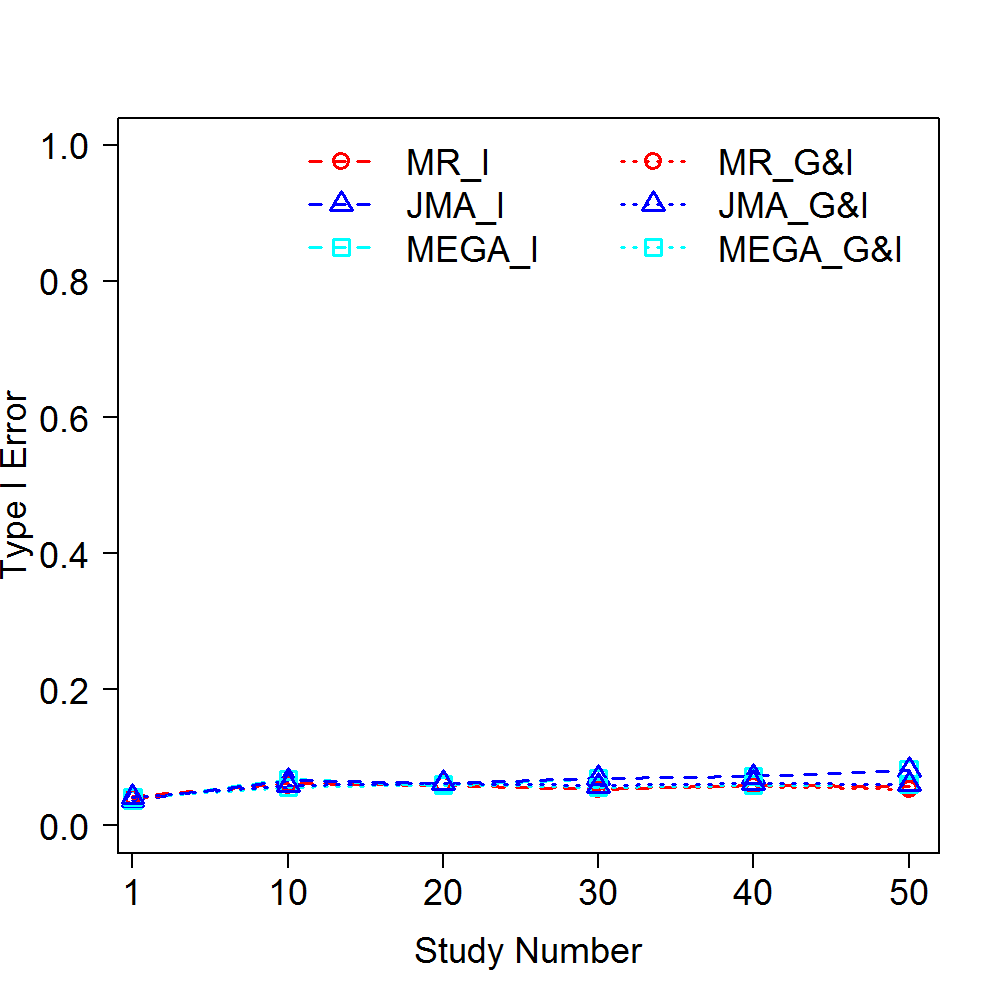

Supplement: S14 Fig — (PNG) [file pone.0171446.s014.png]

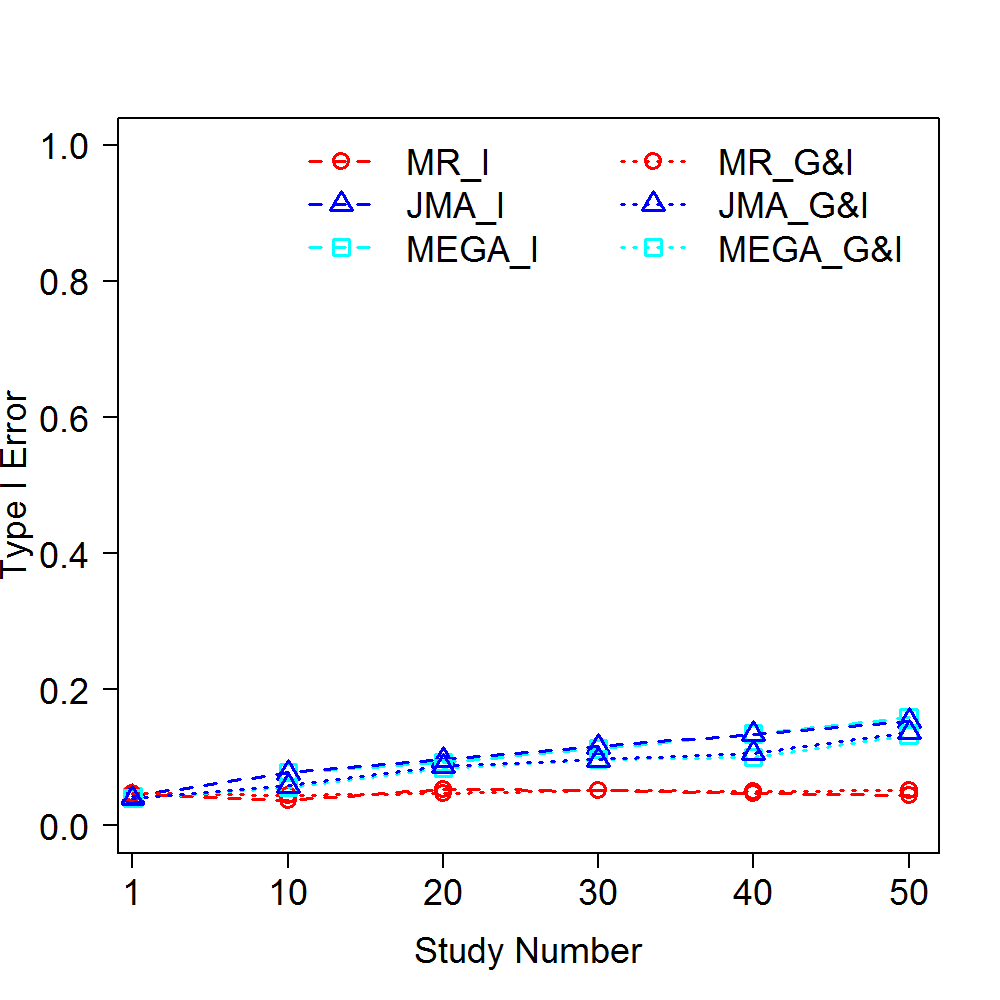

Supplement: S15 Fig — (PNG) [file pone.0171446.s015.png]

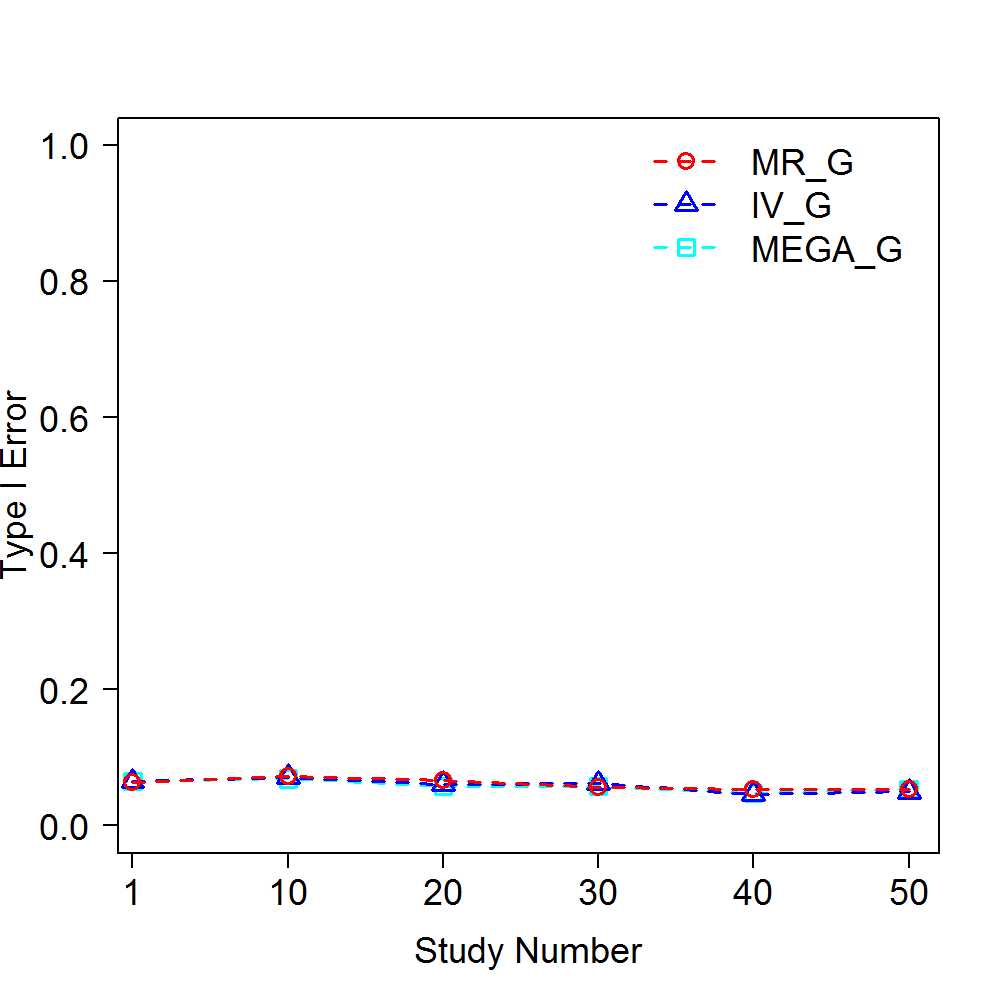

Supplement: S16 Fig — (PNG) [file pone.0171446.s016.png]

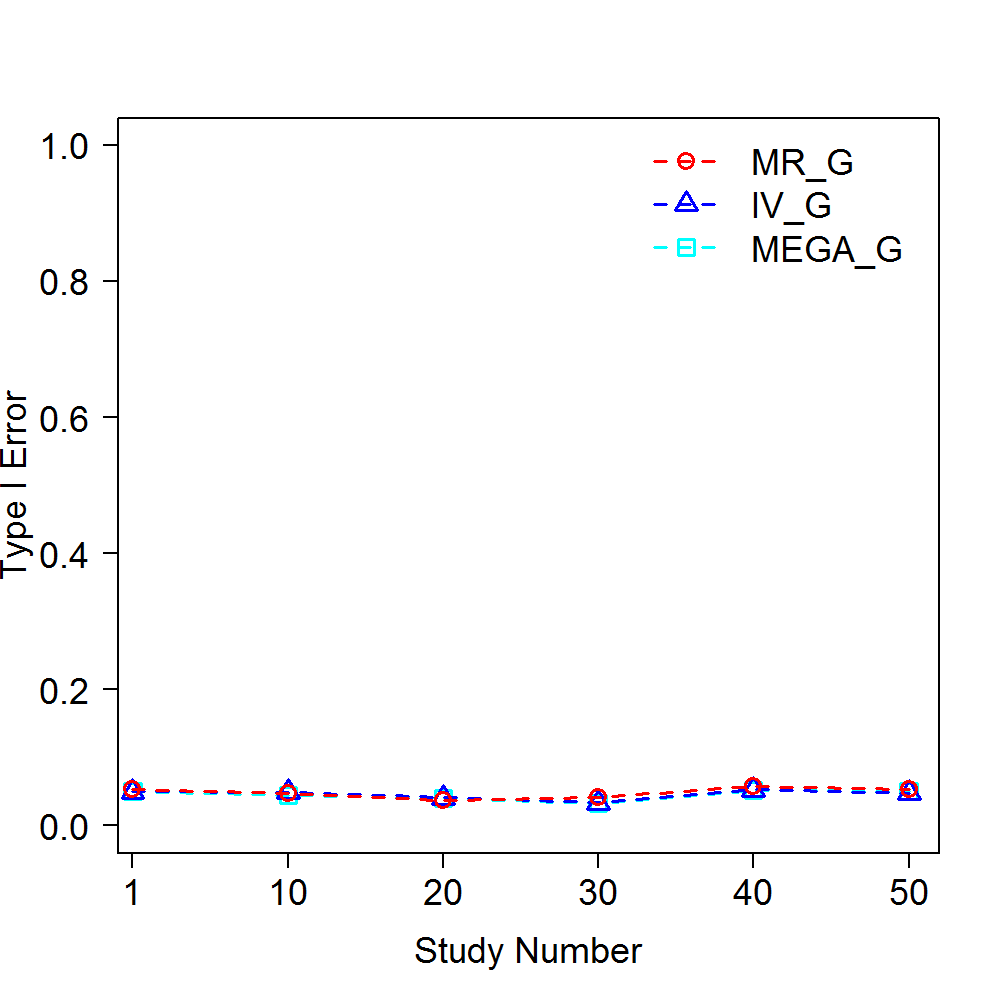

Supplement: S17 Fig — (PNG) [file pone.0171446.s017.png]

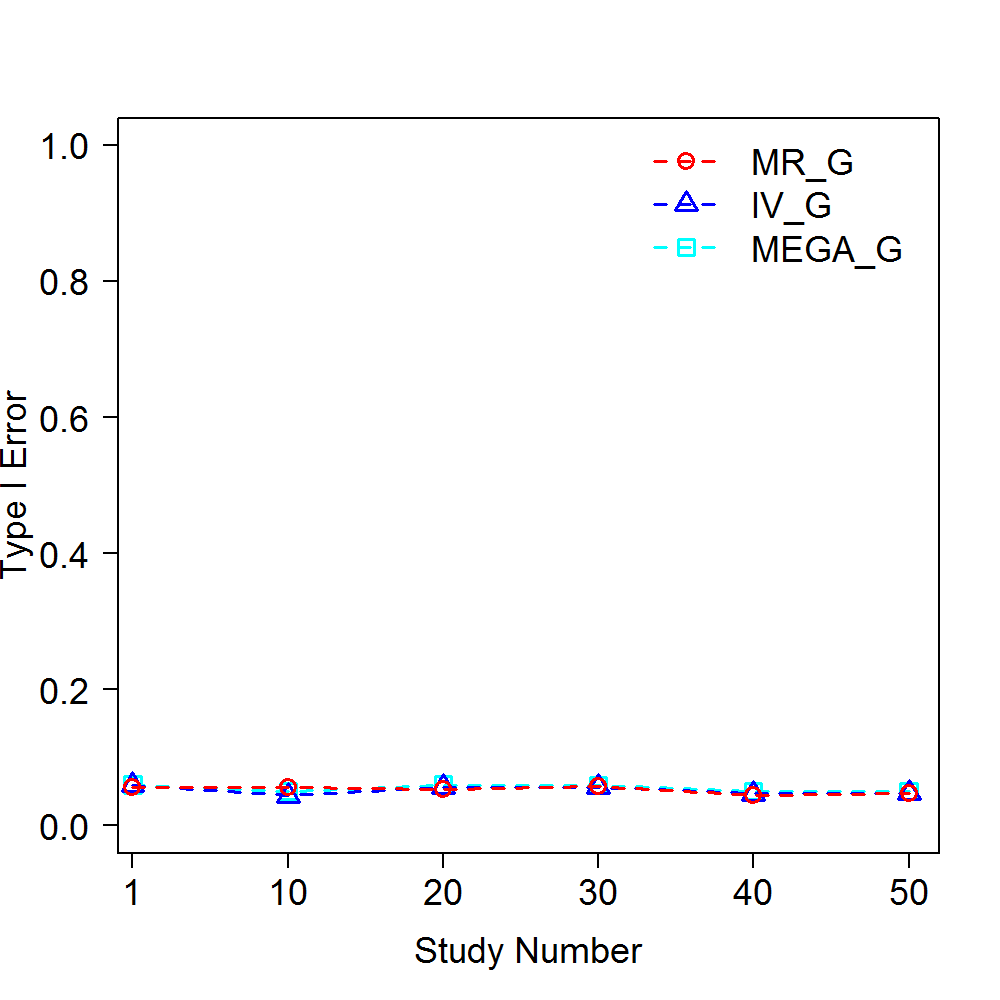

Supplement: S18 Fig — (PNG) [file pone.0171446.s018.png]

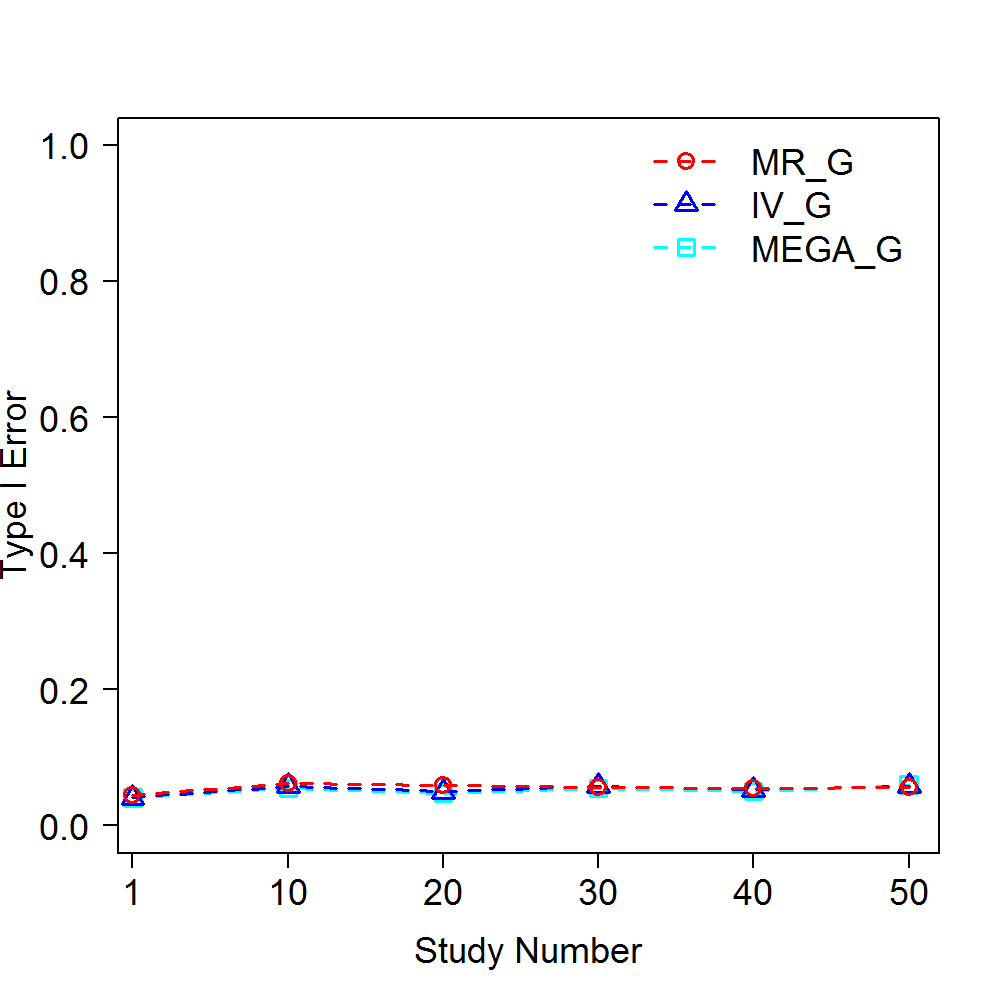

Supplement: S19 Fig — (PNG) [file pone.0171446.s019.png]

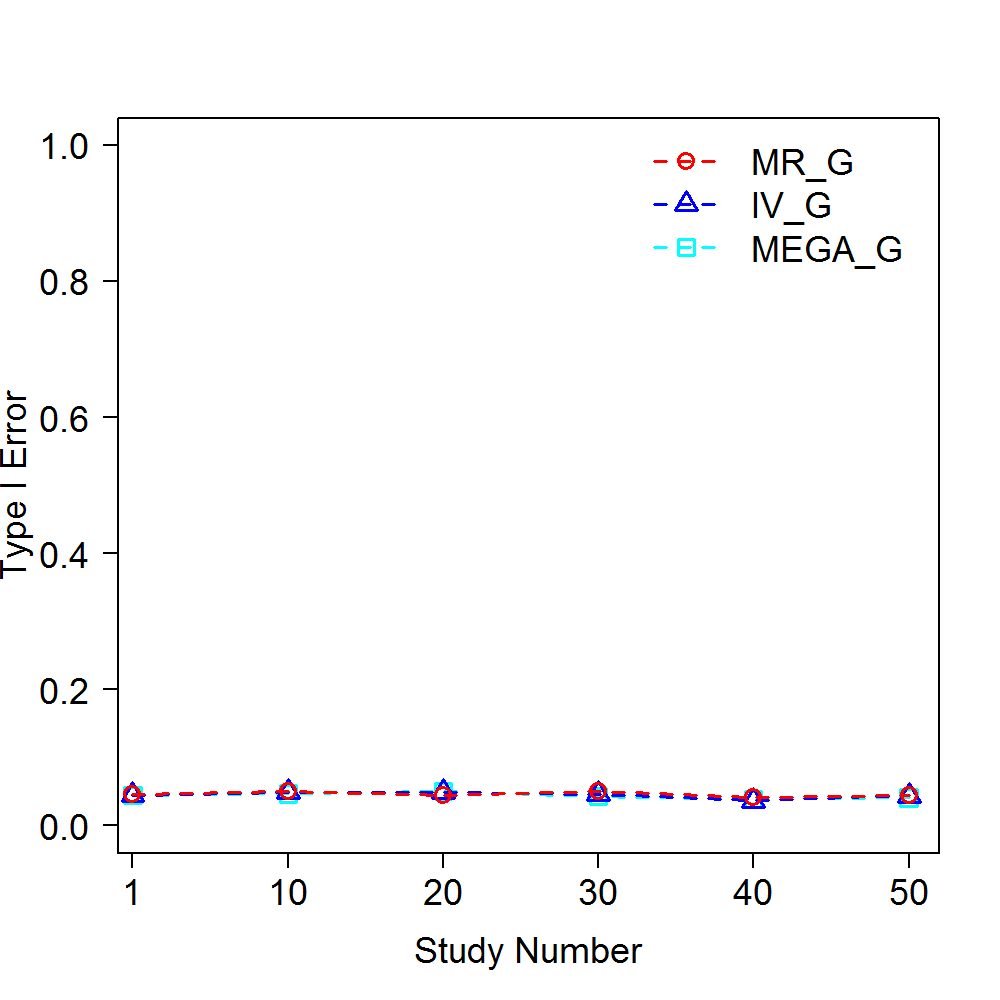

Supplement: S20 Fig — (PNG) [file pone.0171446.s020.png]

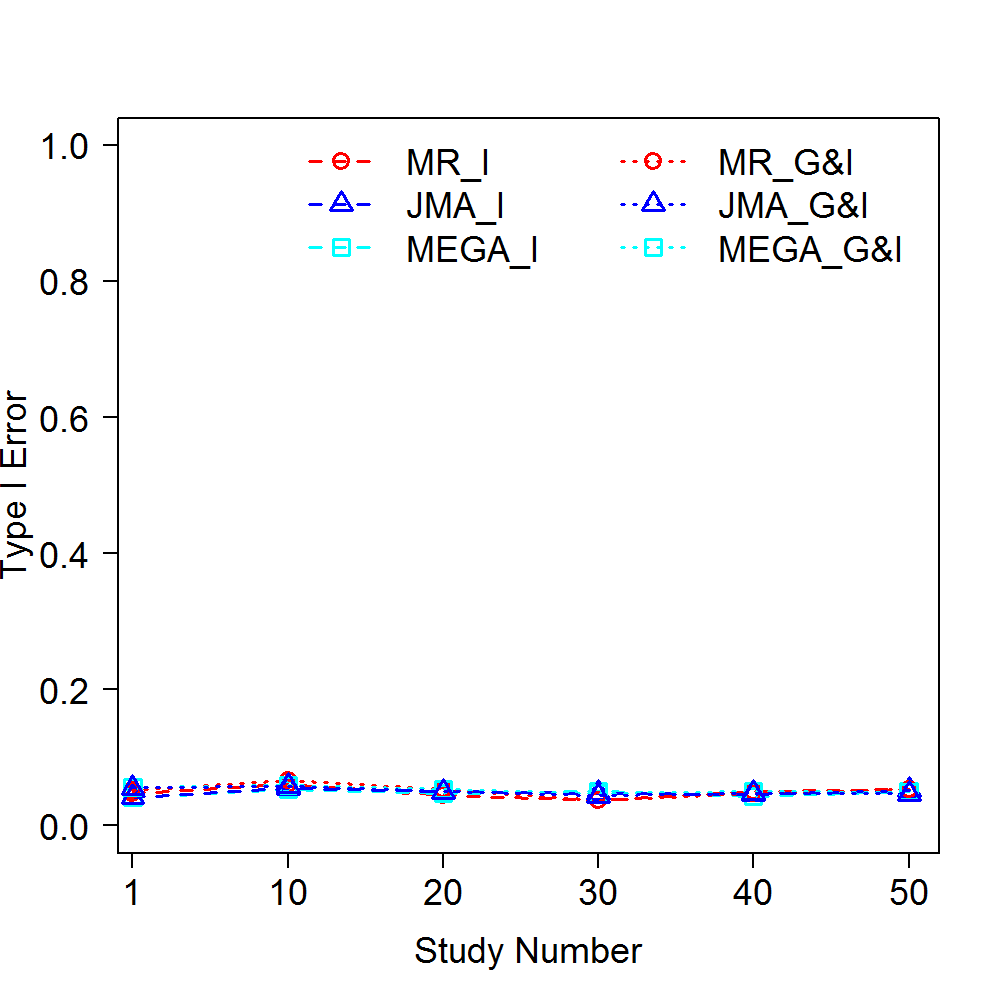

Supplement: S21 Fig — (PNG) [file pone.0171446.s021.png]

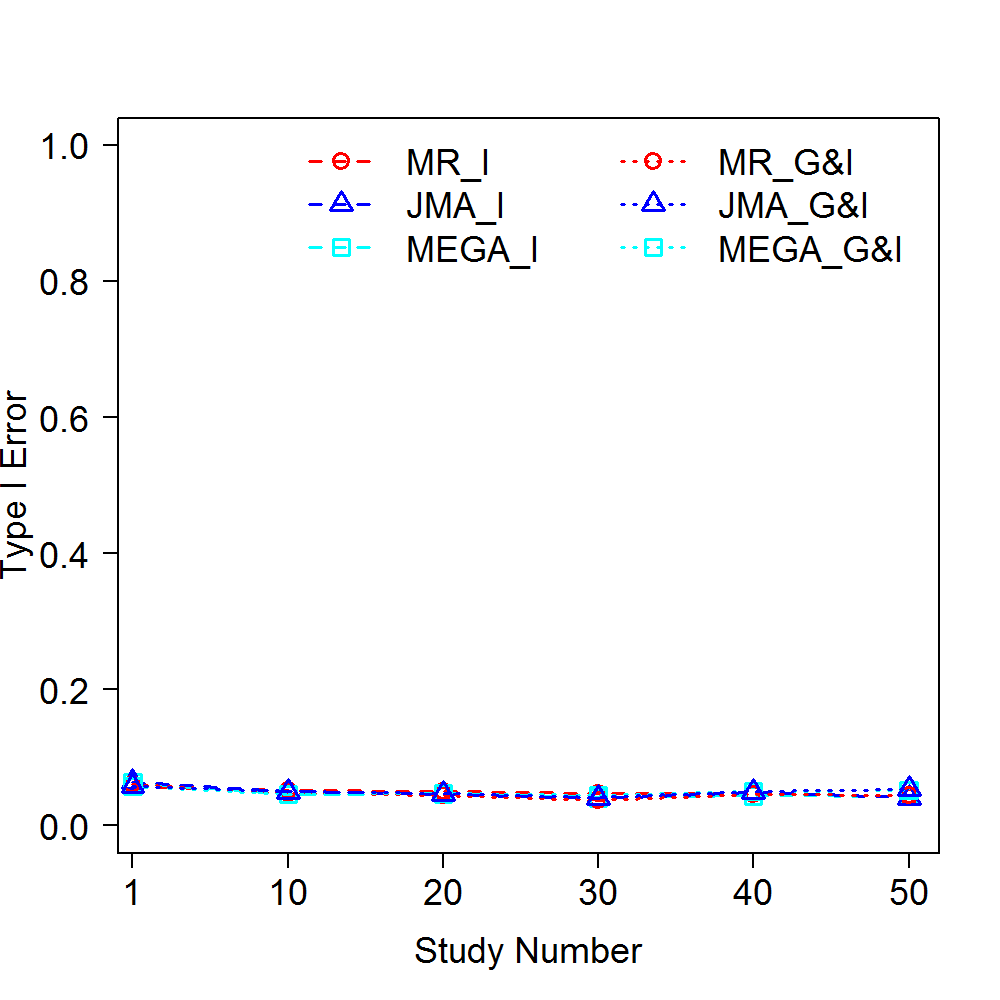

Supplement: S22 Fig — (PNG) [file pone.0171446.s022.png]

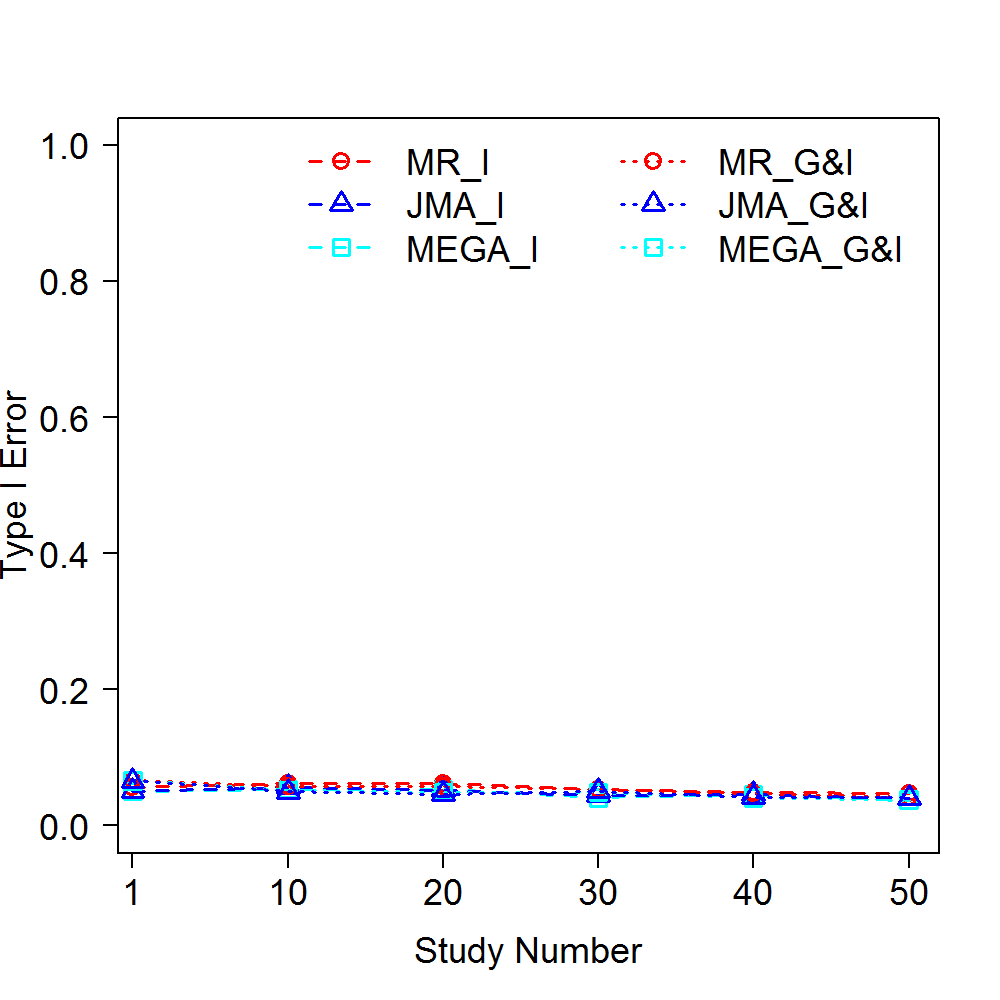

Supplement: S23 Fig — (PNG) [file pone.0171446.s023.png]

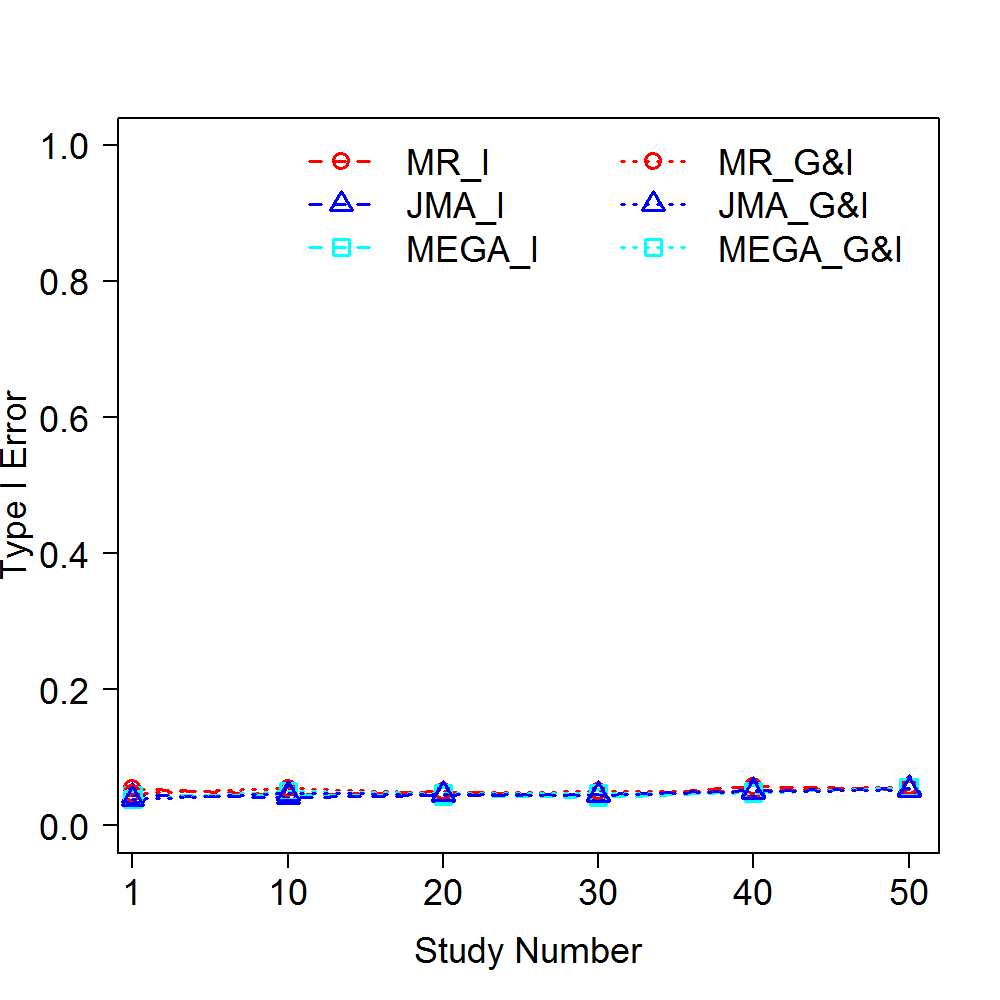

Supplement: S24 Fig — (PNG) [file pone.0171446.s024.png]

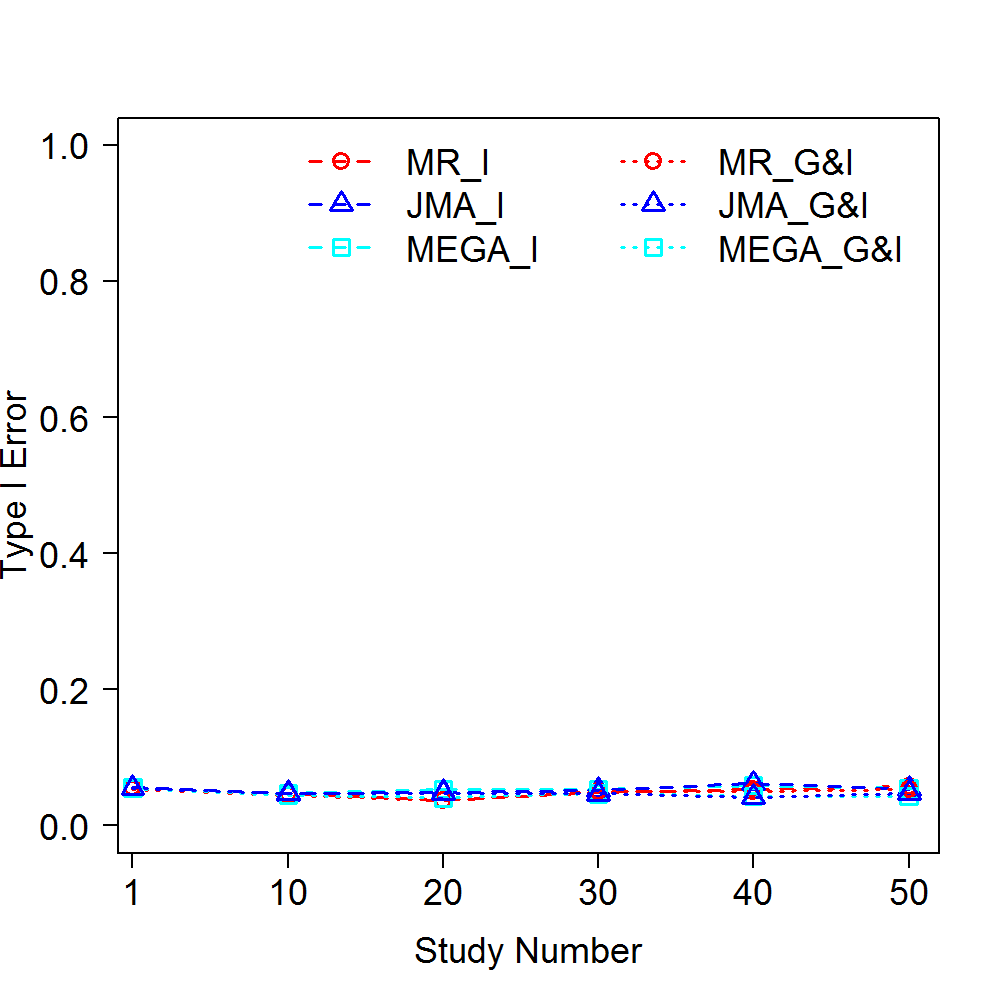

Supplement: S25 Fig — (PNG) [file pone.0171446.s025.png]

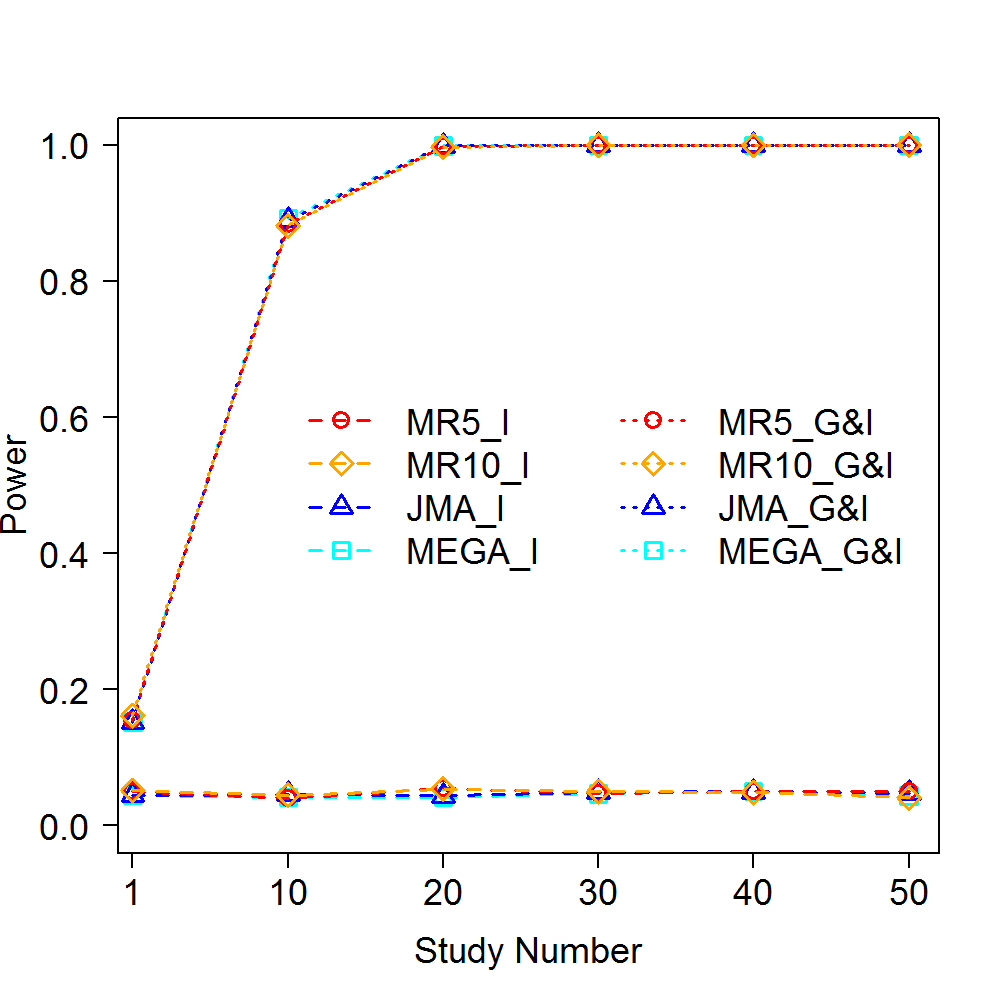

Supplement: S26 Fig — (PNG) [file pone.0171446.s026.png]

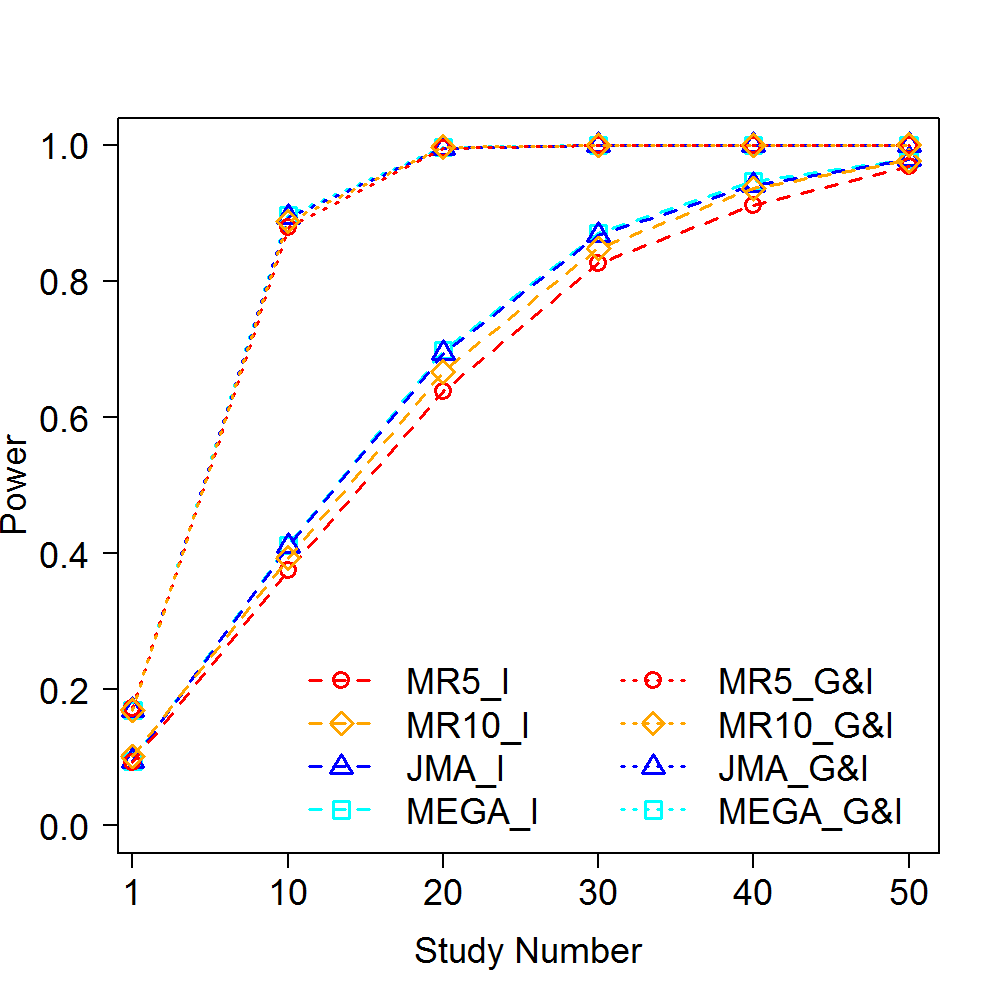

Supplement: S27 Fig — (PNG) [file pone.0171446.s027.png]

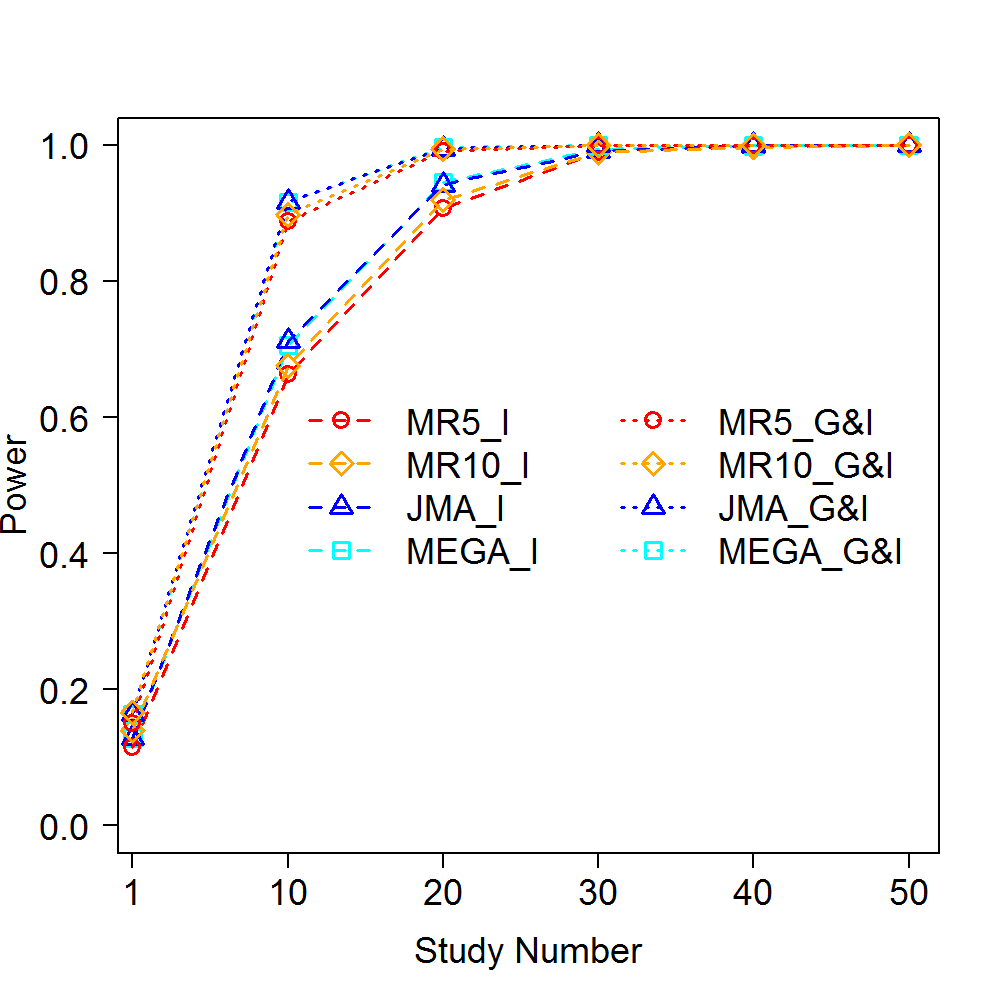

Supplement: S28 Fig — (PNG) [file pone.0171446.s028.png]

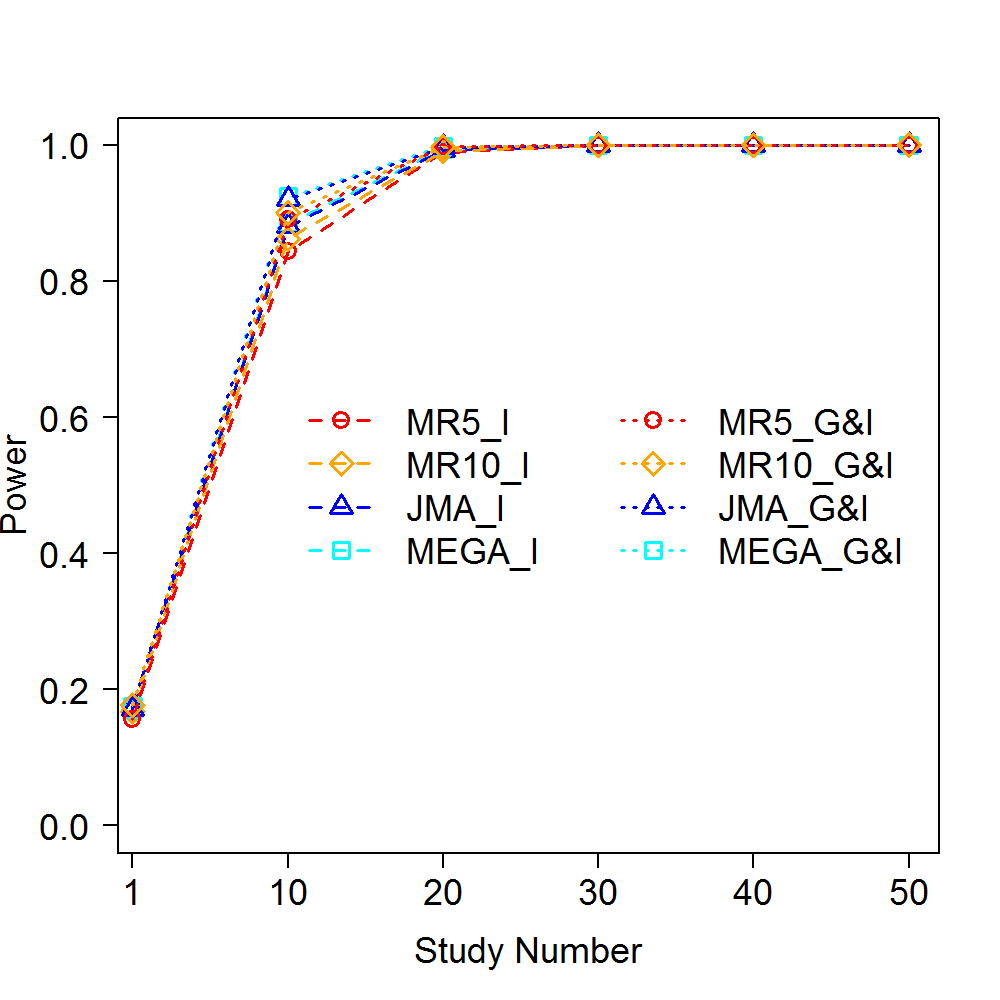

Supplement: S29 Fig — (PNG) [file pone.0171446.s029.png]

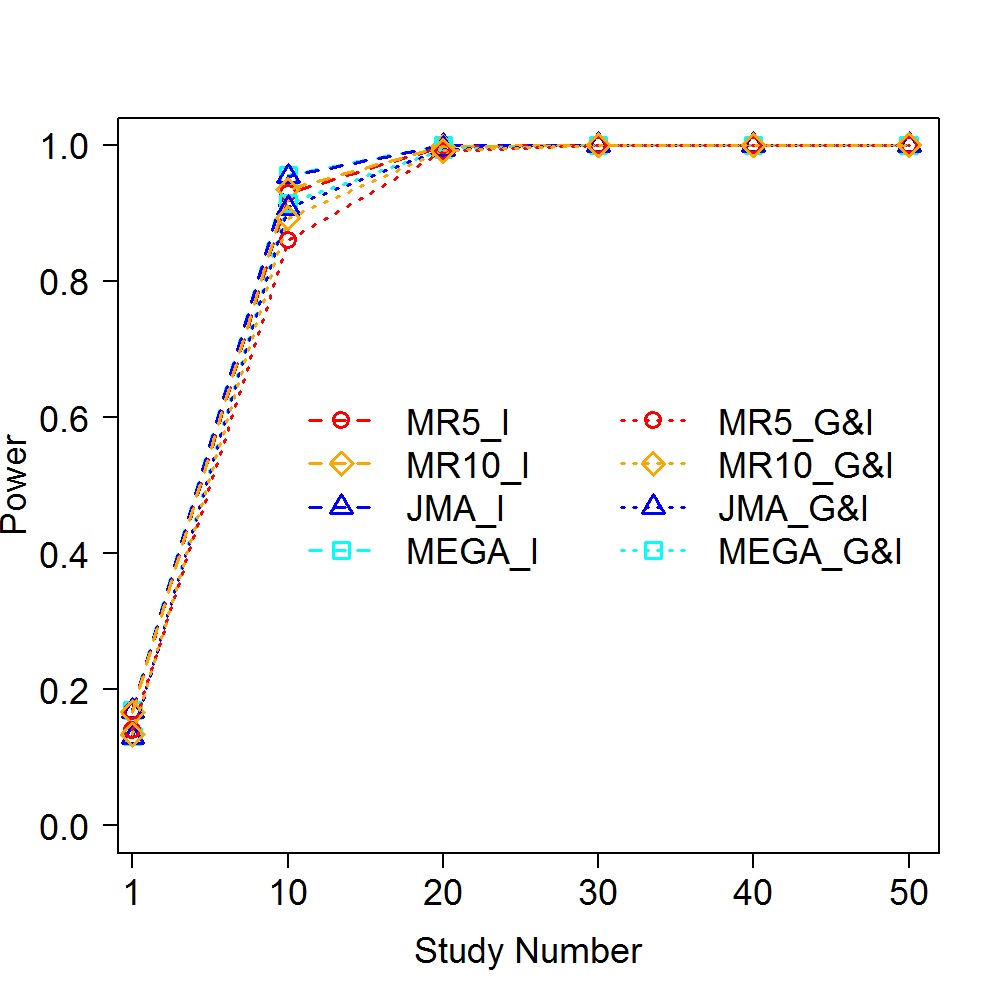

Supplement: S30 Fig — (PNG) [file pone.0171446.s030.png]

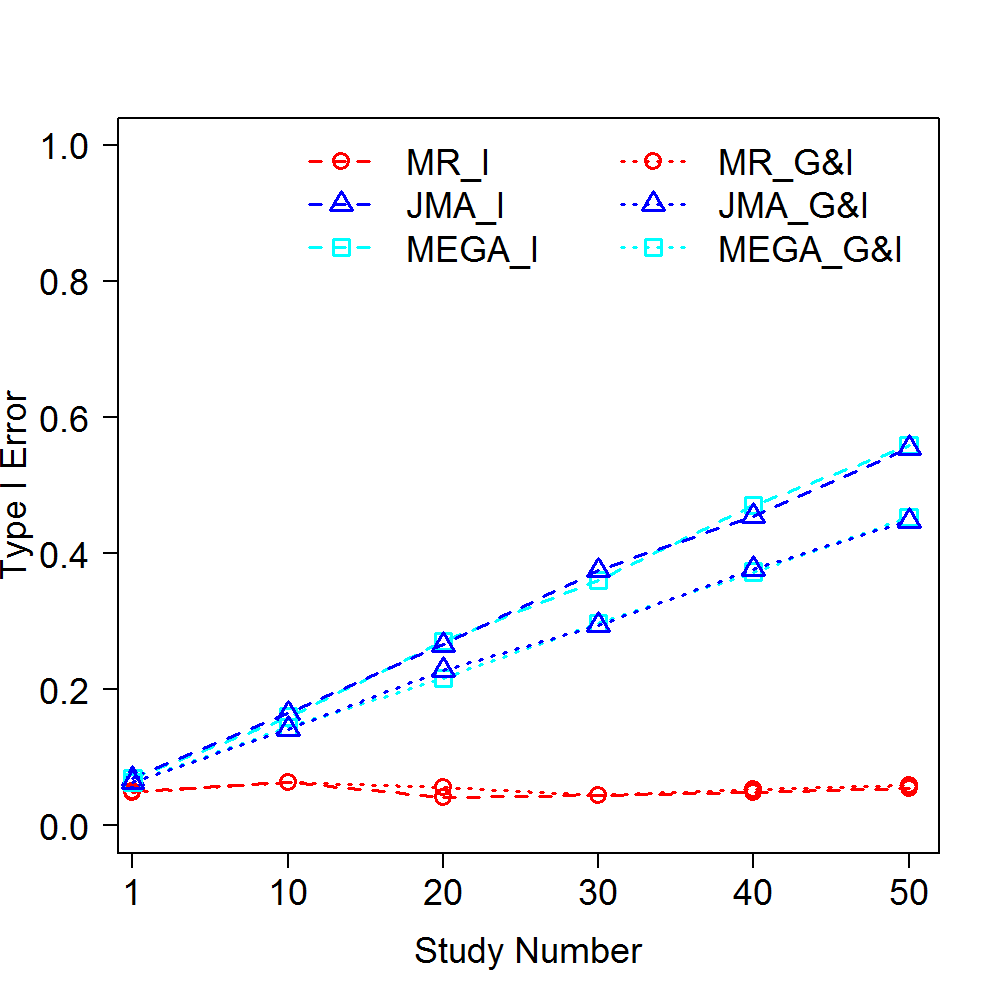

Supplement: S31 Fig — (PNG) [file pone.0171446.s031.png]

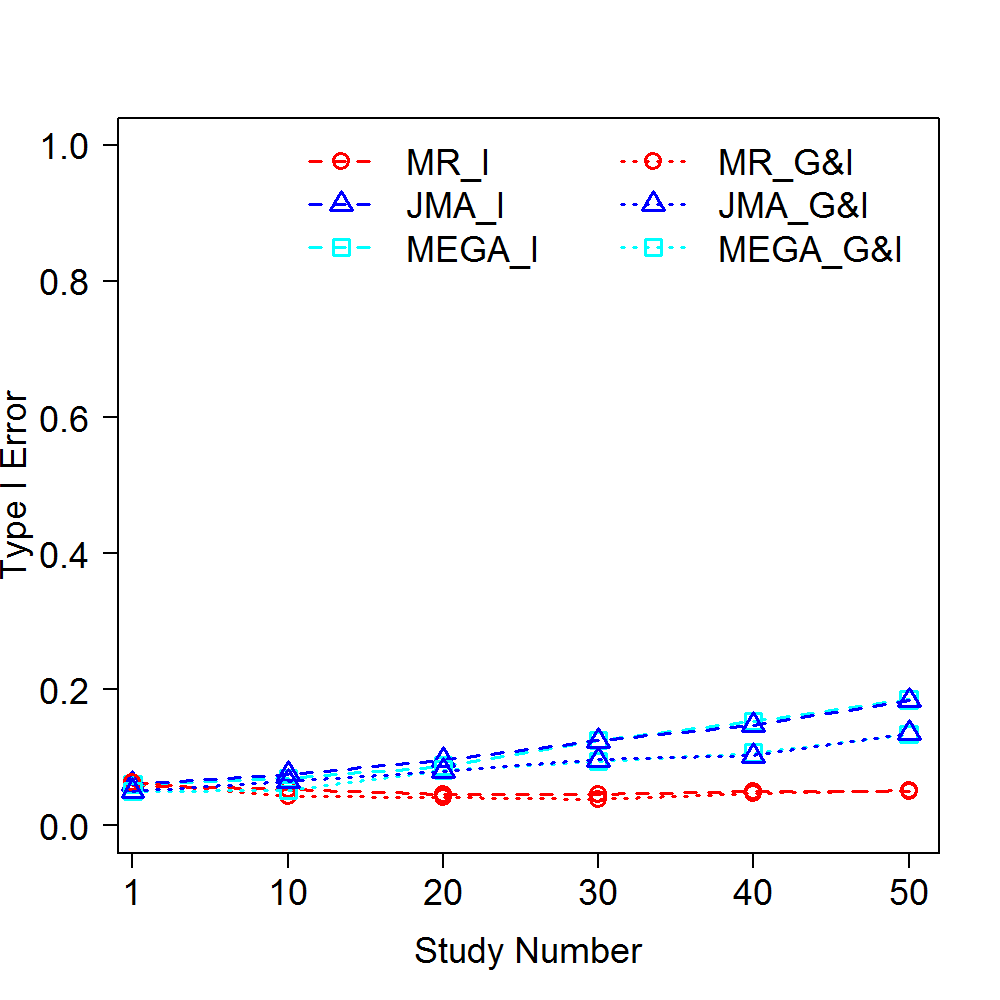

Supplement: S32 Fig — (PNG) [file pone.0171446.s032.png]

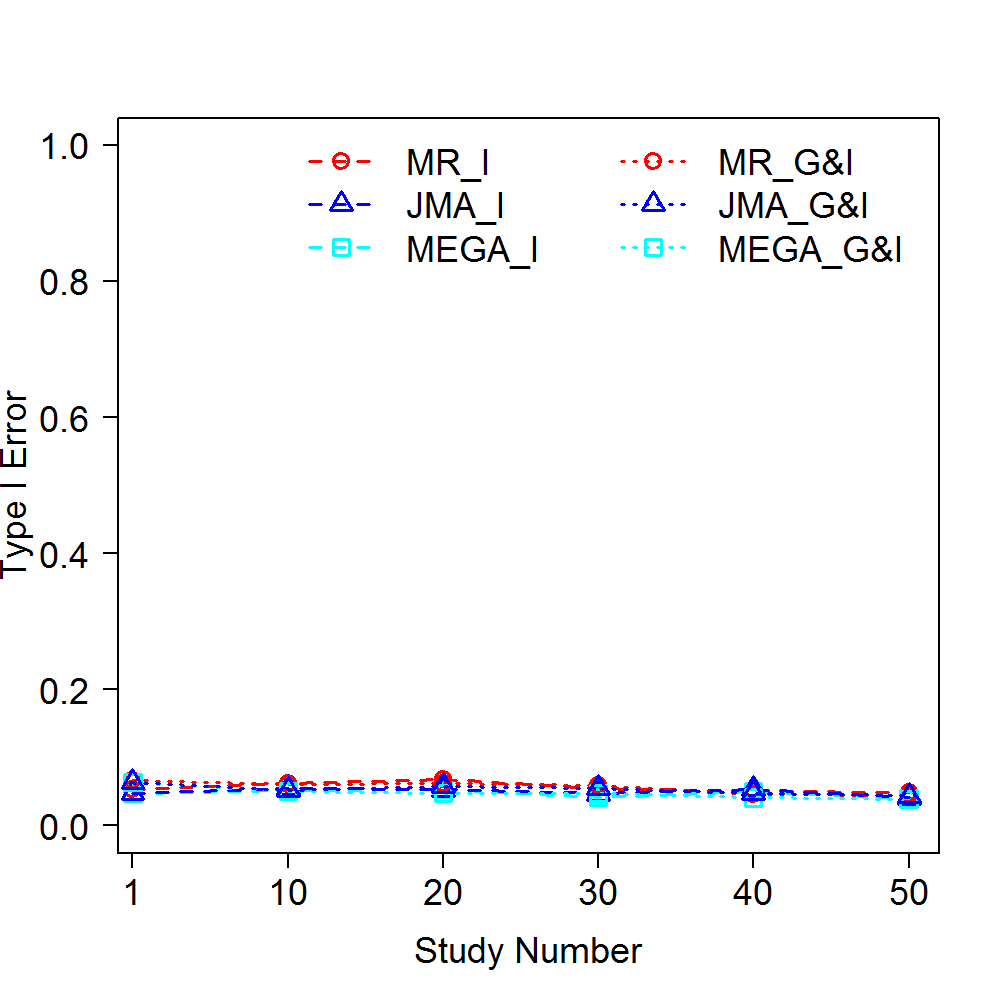

Supplement: S33 Fig — (PNG) [file pone.0171446.s033.png]

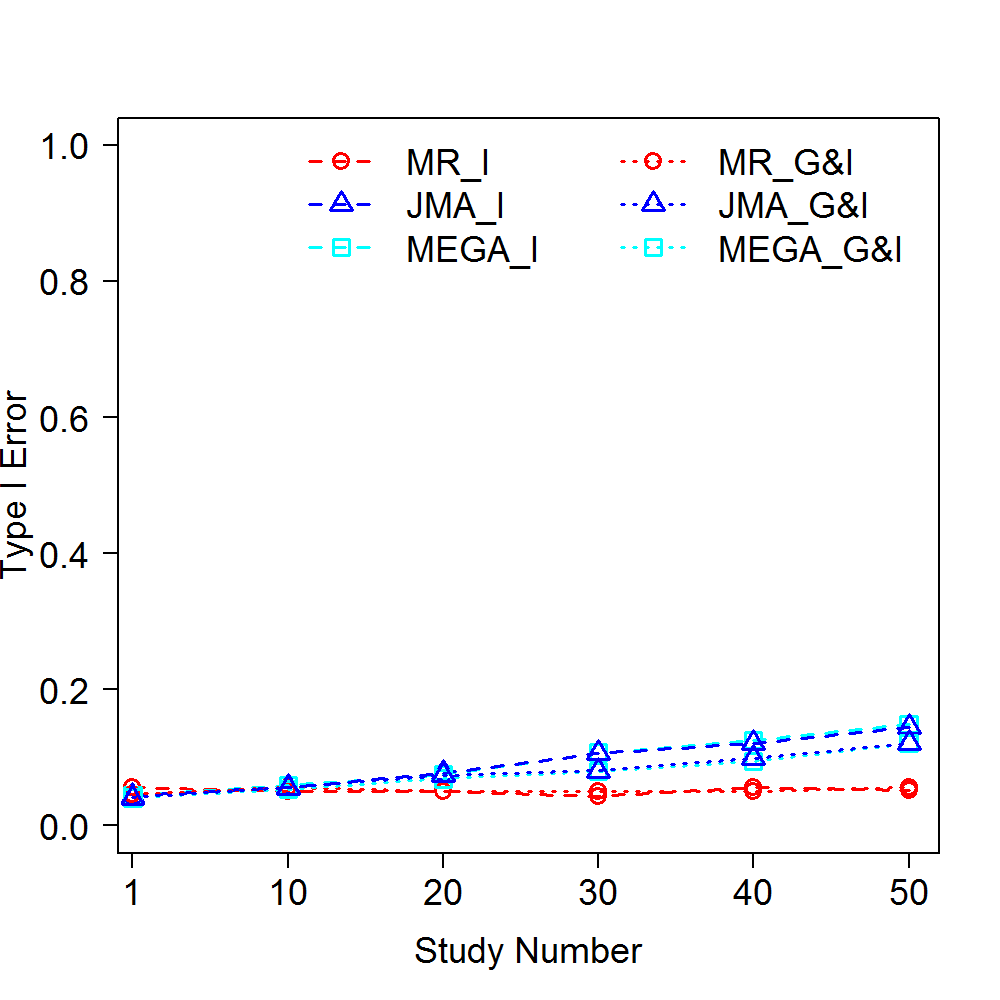

Supplement: S34 Fig — (PNG) [file pone.0171446.s034.png]

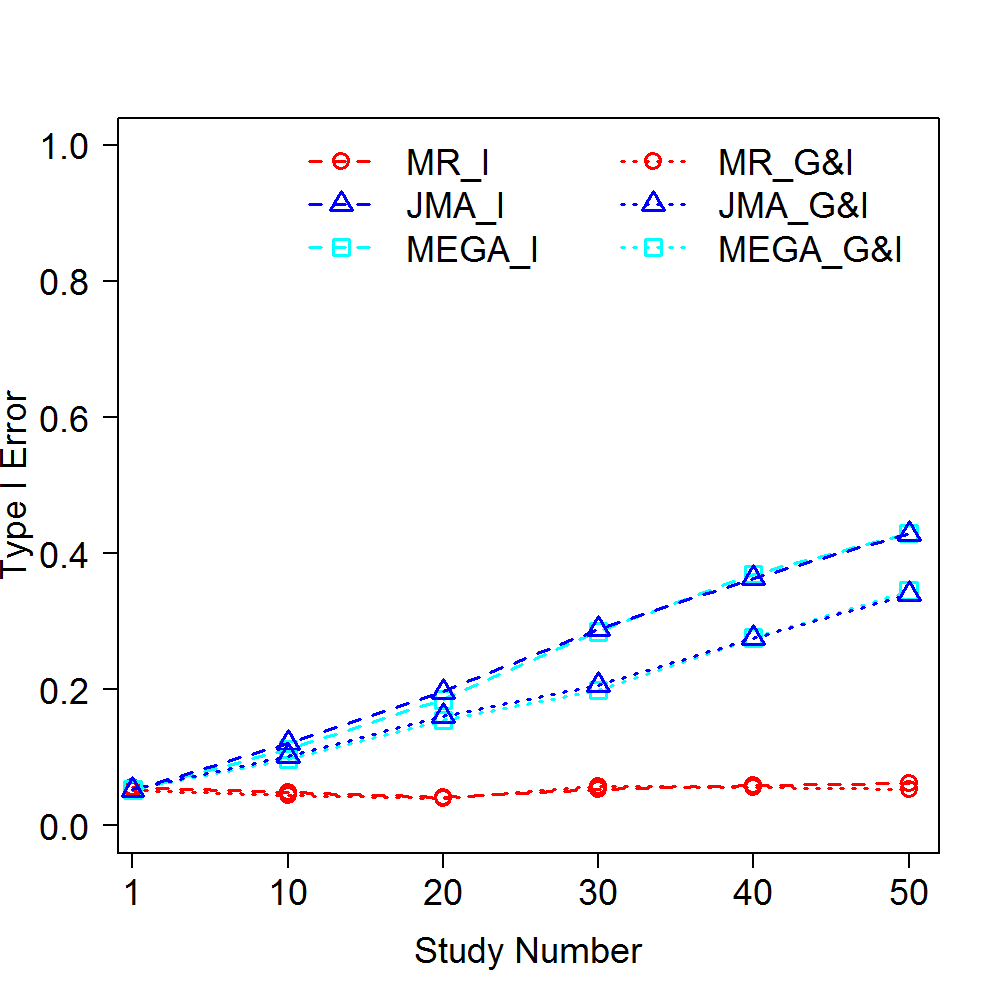

Supplement: S35 Fig — (PNG) [file pone.0171446.s035.png]
